# Creative exploration as a scale-invariant search on a meaning landscape

**DOI:** 10.1038/s41467-018-07715-8

**Published:** 2018-12-21

**Authors:** Yuval Hart, Hagar Goldberg, Ella Striem-Amit, Avraham E. Mayo, Lior Noy, Uri Alon

**Affiliations:** 1 Paulson School of Engineering and Applied Sciences, Harvard University, Cambridge, MA 02138 USA; 2 The Theatre Lab, Weizmann Institute of Science, 76100 Rehovot, Israel; 3 Department of Molecular Cell Biology, Weizmann Institute of Science, 76100 Rehovot, Israel; 4 Department of Neurobiology, Weizmann Institute of Science, 76100 Rehovot, Israel; 5 Department of Psychology, Harvard University, Cambridge, MA 02138 USA; 6 Department of Psychology, Ben Gurion University of the Negev, 8410501 Beer-Sheva, Israel

**Keywords:** Computational neuroscience, Scale invariance, Cognitive neuroscience, Systems biology

## Abstract

Can knowledge accumulated in systems biology on mechanisms governing cell behavior help us to elucidate cognitive processes, such as human creative search? To address this, we focus on the property of scale invariance, which allows sensory systems to adapt to environmental signals spanning orders of magnitude. For example, bacteria search for nutrients, by responding to relative changes in nutrient concentration rather than absolute levels, via a sensory mechanism termed fold-change detection (FCD). Scale invariance is prevalent in cognition, yet the specific mechanisms are mostly unknown. Here, we screen many possible dynamic equation topologies, to find that an FCD model best describes creative search dynamics. The model further predicts robustness to variations in meaning perception, in agreement with behavioral data. We thus suggest FCD as a specific mechanism for scale invariant search, connecting sensory processes of cells and cognitive processes in human.

## Abstract (web summary)

Molecular circuits implementing fold-change detection (FCD) allow cells to respond to fold-change in signals regardless of absolute levels. Here, the authors find that meaning, attention and saturation similarly form an FCD circuit and produce the observed dynamics of human behavior in creative search.

## Introduction

Symmetries and invariances are powerful concepts in physics^1–4^. Seeking invariances has also been fruitful in biological systems, especially in the study of how cells make sense of their environment. For example, in order to search for food, *E. coli* climbs gradients of nutrients using a sensory mechanism that has scale invariance, responding to the fold-change in inputs rather than to their absolute level^5–7^. This scale invariance is known as fold-change detection (FCD): the dynamic response (including amplitude and response time) is invariant to multiplying the input by a scalar. Thus, the dynamics to a step of signal from 1 to 2 is identical to a step from 2 to 4, because both have twofold-change. FCD allows bacteria to optimally find maximal nutrient levels despite varying level of attractant sources (the source strength multiplies the input field by a constant, and FCD normalizes this constant out)^8^. FCD mechanisms appear in diverse cell signaling pathways^9^, as well as in human sensory systems such as vision and hearing, explaining the well-known Weber-Fechner and Stevens laws^10,11^ in which response is scaled to the background signal.

FCD combines two general features of sensory systems: exact adaptation in which the output returns to a baseline level that is independent on background input signal, and Weber’s law, in which the response amplitude depends on the relative change in input and not the absolute change. FCD is more restrictive than Weber’s law, since it requires the entire dynamic response—including amplitude and response time—to depend only on relative changes in input. Thus, FCD makes search dynamics robust to a global change of scale^5^. FCD is a constraint that limits the possible mechanisms at play to a small class of circuits with mathematical homogeneity properties^7,12,13^. In fact, only two main classes of circuits - an incoherent feedforward loop and a nonlinear integral feedback loop - provide FCD in known cases.

Based on its success in cells, one can ask if FCD mechanisms can also help us understand human cognitive behavior. Scaling invariance has been suggested to occur widely in cognition—where people’s behavior is nearly identical across several orders of magnitude. Consequently, scale invariance was suggested as a shared principle for human behaviors^14,15^ such as perception^16,17^, memory^18–20^, decision making^21^, reaction times^22,23^, motor control^23–25^, and language generation^26^. For example, Brown and Chater discussed the prevalence of scale invariance in perceptual mechanisms^16^ which yields Weber-Fechner and Stevens laws. They later developed theories for memory dependence on timing and decision based on sampling that produce similar scale invariance behaviors in experiments^19,21^. Other studies have shown that scale invariance emerges also in neural activity on the network level^27,28^ and in specific areas such as the dopamine neurons of the reward system^29–31^.

One explanation of scale invariance in neural and behavioral data portrays the cognitive process as a dynamical system at the edge of criticality, where correlations across orders of magnitude yield power-law-like behavior seen in scale invariant systems. It would be important to complement this view with mechanisms similar to biological physics, which employ feedforward and feedback loops circuits^5,32^, with no need to invoke criticality arguments.

**Fig. 1:**
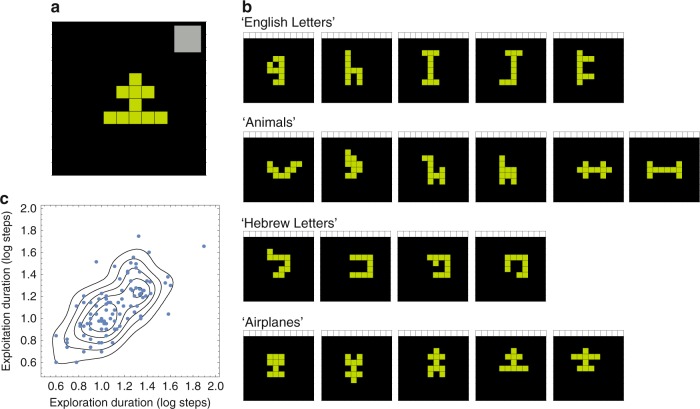
The creative foraging game as a paradigm to study creative search. **a** A schematic of the creative foraging game screen that players use to search for shapes. Each step is a move of one square, keeping the ten squares connected. Players can choose shapes to a gallery (previously chosen gallery shape is presented in the gray square area at the top right). **b** In exploitation phases, players typically collect shapes from distinct visual categories (e.g., ‘English Letters’, ‘Animals’, ‘Hebrew Letters’, ‘Airplanes’, etc…). Each row of shapes is one exploitation phase of a player in the game. **c** Variation between players shows a high correlarion between each player's median durations of exploration and exploitation phases. Blue points indicate players’ median search durations in the exploration-exploitation duration plane (in log steps). Topographic map indicates lines of equal density of exploration-exploitation durations

Here, we test for FCD mechanisms in a human cognitive task, creative search. We analyze experimental data on high-resolution measurements of behavior and individual differences in the creative foraging game (CFG) task^33^. CFG is an online game in which players search a space of geometric shapes made of ten connected squares. Players create shapes made of 10 connected squares, moving one square at a time, to find new shapes (Fig. 1a). In their search, players are asked to collect ‘interesting and beautiful’ shapes to a gallery (Fig. 1a). We recorded players’ entire trajectories including every square move, its timing, and the gallery choices they made^33,34^.

The creative foraging game showed the following salient features. (i) Players’ trajectories consisted of two phases—exploration and exploitation. (ii) In each exploitation phase, players collect shapes from a distinct visual meaning category (e.g., ‘English letters’, ‘Digits’, ‘Animals’, etc…). Different players find similar categories in their search (Fig. 1b). (iii) Players leave an exploitation phase long before the category is depleted, to start a meandering exploration phase. Exploration ends when a new category is discovered, initiating a new exploitation phase, and so on. (iv) Players’ exploration and exploitation durations were highly correlated: players with long exploration also had long exploitation phases. People thus varied along a one-dimensional continuum between a mercurial strategy of quick-to-discover a new category but quick-to-drop the category, and a more thorough slow-to-discover/slow-to-drop behavior (Fig. 1c).

Known search processes such as simulated annealing and naive Bayesian search cannot account for such patterns. Here, we asked what mechanism can underlie such behavioral results, and suggest a way to understand human search in this game using FCD concepts.

## Results

### The game induces multi-layered meaning landscapes

To understand creative search dynamics, we start by constructing a meaning landscape on the network of shapes. The network of shapes describes all 36,446 shapes made of ten squares. Each node is a shape, and edges connect shapes that can be reached by moving one of the squares. Shapes have a median of 57 neighbors. Players moved on this network of shapes, one edge at a time, during the game.

**Fig. 2:**
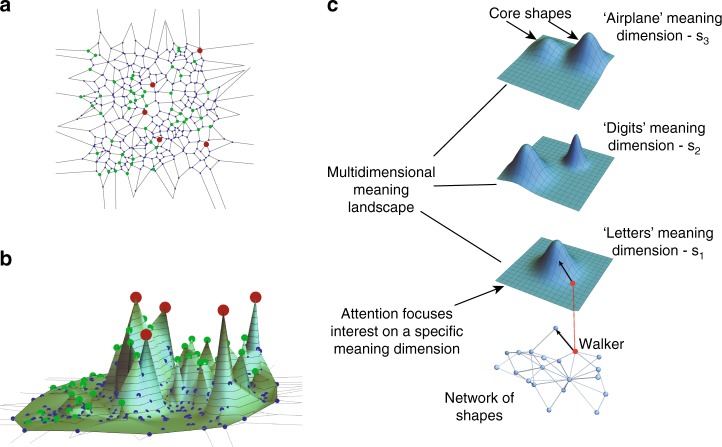
Players’ dynamics induces multi-layered meaning landscapes. **a** A searcher walks on the network of shapes. The network of shapes consists of core shapes (marked in red), gallery shapes (marked in green) and all other shapes (marked in blue). Shapes are connected by one move of a square. **b** The meaning landscape built on top of the network of shapes. Close by core shapes typically belong to different meaning categories. **c** The meaning landscape is composed of a multi-layered space of meaning dimensions. Each shape has M dimensions of meaning, corresponding to M meaning landscapes which show high meaning for shapes in the core of the corresponding category (‘airplanes’, ‘digits’, ‘letters’ etc…). The attention vector focuses the searcher to do hill-climbing along a specific meaning dimension

In order to form models of the search process, we began by assigning meaning to each shape. We assigned to each shape a meaning vector ${\vec{\mathbf{s}}}$ with M dimensions. Each dimension corresponds to one of the six categories most commonly found by players (Fig. 2). For example, **s**_1_ is the ‘English letters’ meaning dimension, **s**_2_ is the ‘Digits’ dimension and so on. The categories were defined in ref. ^33^ by clustering shapes found in exploitation phases by different players.

To define the meaning landscape, we assigned high-meaning value **s**_i_ to shapes in a meaning category i that were discovered by many players (meaning value is proportional to the number of players that found the shape). We call these shapes core shapes. For example, core shapes in the digit category resemble prototypical digits 5, 7, 9 and so on. The meaning assigned to other shapes decays exponentially with their distance along the network from the core shapes (Fig. 2a, b and see Methods and Supplementary Information). Shapes close to two shapes with meaning categories i and j are thus assigned meaning in both meaning vector components **s**_i_ and **s**_j_. Shapes far from any core shape have low meaning in all dimensions (Fig. 2 and Supplementary Fig. 1).

### Brain activity responds to relative meaning score induction

We next assessed the correlation of human brain activity with the present meaning landscape structure. In a functional imaging experiment^35^ (fMRI, see Methods), we showed shapes with varying meaning scores to 15 subjects while scanning their brain activity (fMRI, see Methods and Goldberg et al.^35^).

**Fig. 3:**
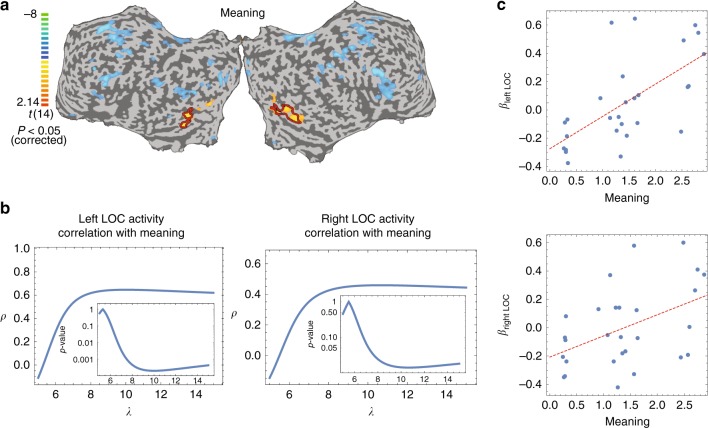
Brain activity correlates with a fold decay of the meaning across shapes’ network. **a** Parametric mapping. Cortical activity maps of multi-subject, random effect, parametric GLM analysis of meaning presented on an unfolded cortex (*N*= 15), map is corrected for multiple comparisons, *p*< 0.05. Color scale indicates *t-*values. Yellow-orange scale represents regions, which showed positive parametric relation with meaning scores (A non-parametric, *t*-max correction analysis to control for FWER showed a *t*-value of 1.7 for *α* = 0.05, see Methods). Blue-green scale represents regions which showed negative parametric relation with meaning scores. **b** Analysis of brain activity correlation strength (Inset, *p*-value) as a function of the decay factor from a core shape (*x-*axis–decay factor *λ*, where meaning decays by *λ*^-*d*^ where *d* is the distance on the network of shapes). Maximal correlation and *p*-value are attained at a fold decay of about *λ* = 10 (see Supplementary Information for linear and power-law decay functions analysis). **c** Correlations of LOC activity and meaning. Scatter plots present the relation between the averaged meaning of blocks (*x-*axis) and the averaged brain activity (*N* = 15, normalized beta weight, *y-*axis) in bilateral LOC. Each dot represents one block. Spearman correlation; left LOC, *r* = 0.65, *p* < 0.0002. right LOC, *r* = 0.48, *p* < 0.02

A parametric correlation analysis between brain activity and the meaning score of shapes showed that specific areas in the visual regions, most predominantly the lateral occipital cortex (LOC), activate parametrically with the meaning score (Fig. 3a). In a parallel study, Goldberg et al.^35^ asked subjects to rate these shapes according to their iconicity value. This independent score, ranked by participants of the brain study, showed high correlation with the meaning score built from players of the creative foraging game (Spearman correlation, *r* = 0.83, *p* < 10^−5^). Moreover, the parametric correlation of brain activity with the iconicity score showed high overlap with the meaning score parametric activity map (Supplementary Fig 2a, see ref. ^35^). Thus, our meaning landscape correlates with LOC activity of people when presented with discovered shapes.

The brain activity measures allowed us to further ask what the best function for induction of meaning between neighboring shapes is, an exponential decay or a linear decay. In the construction of the meaning landscape we used an induction function of meaning that decays in a constant rate with each distance unit (i.e., meaning decays exponentially with distance *d* as *λ*^*−d*^ and meaning is measured on a relative scale), but other decay functions are possible as well. To that aim, we repeated the procedure of meaning landscape construction with a linear decay function (i.e., meaning decays with distance by –*λd*, hence meaning is measured on an absolute level). We find that brain activity is best explained by the exponential decay model with a decay rate of about *λ* = 10 (see Fig. 3b, Methods). With *λ* = 10, beta activity in the LOC area correlates sizably and significantly with the meaning score of shapes’ blocks (Spearman correlation, left LOC, *r* = 0.65, *p* < 0.0002. right LOC, *r* = 0.48, *p* < 0.02, Fig. 3c). These findings suggest that LOC brain activity responds to relative changes rather than absolute levels of meaning induction. What mechanism might then explain people’s creative search on this meaning landscape?

### Creative search model with attention and saturation variables

We next built dynamic models of the search process on the network. Our goal is to capture the basic experimental observations (features (i)–(iv) above): existence of exploration-exploitation phases, collection of shapes from the same meaning category in each exploitation phase, dropping of a category far before it is depleted to search for new categories, and variation between players along a mercurial-thorough continuum.

In the model, an agent walks on the network of shapes, seeking to climb gradients of the meaning landscapes. To avoid being stuck on a local maximum, the walk is self-avoiding.

We begin with a naive hill-climbing model in which the searcher moves to the neighbor with highest meaning in any dimension. The next shape ${\vec{\mathbf{s}}}(t + 1)$ is thus

1 $${\vec{\mathbf{s}}}\left( {t + 1} \right) = {\mathrm{arg}}\,{\mathrm{max}}_{s \in s\left( t \right){\ }{\mathrm{Neighbors}}}{\vec{\mathbf{s}}}\left( t \right)$$

**Fig. 4:**
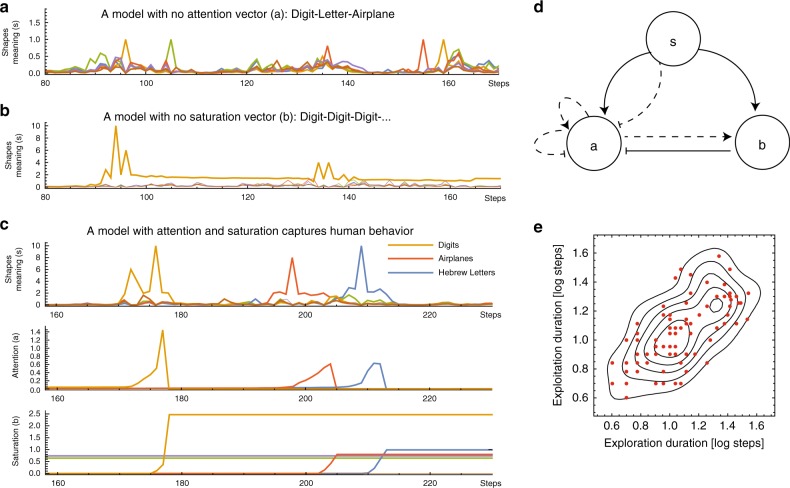
Attention and saturation variables capture behavioral exploration-exploitation. **a** Search with no attention jumps between categories. **b** Search with no saturation is stuck at a single category. **c** Search with both attention and saturation variables can produce human-like search. **a**–**c** Line colors represent different meaning dimensions. **d** The general circuit for creative search—shapes with meanings induce attention in a specific meaning dimension to grow, attention (**a**) can increase its own levels, inhibit attention in other meaning dimensions, and above a certain threshold attention levels increase saturation (**b**) which in turn, inhibits attention. Solid lines are obligatory connection of the circuit. Dashed lines mark possible connections between variables. **e** An FCD circuit (see Eqs. (5) and (6)) with different parameters (red dots) captures similar dynamics of the exploration-exploitation plane as the behavioral data (marked by lines of equal density of players’ exploration-exploitation durations). The correlation of the simulated data is similar to the behavioral data (FCD: Spearman correlation, *r* = 0.71, *p* < 10^−5^, Behavioral data: Spearman correlation, *r* = 0.73, *p* < 10^−5^)

This model disagreed with the experiment: Instead of finding a sequence of shapes from the same category (e.g. digit, digit, digit) before moving to a new category, the hill-climbing model resulted in sequential shapes from different categories (e.g. letter, airplane, digit), etc. (Fig. 4a). It thus had no exploitation phases focused on a single category. The failure is due to the fact that highest meaning shapes are interleaved on the network of shapes: close to a shape with high meaning in one category are high-meaning shapes from other categories.

The agent thus requires a mechanism to stay in one category. To supply this, we added an attention variable, **a**_i_(*t*), to the agent, to focus moves to a single meaning dimension once a meaning threshold is crossed. If attention is below threshold *T*_1_, a random neighbor is chosen. If attention in a certain dimension crosses the threshold, the shape with most meaning in that dimension ${\vec{\mathbf{s}}} \cdot {\vec{\mathbf{a}}}$ is chosen

2 $${\vec{\mathbf{s}}}\left( {t + 1} \right) = \left\{ {\begin{array}{*{20}{l}} {{\mathrm{Random}}\,{\mathrm{neighbor}}} \hfill & {\max \,\overrightarrow {\bf{a}} \left( t \right) \le T_1} \hfill \\ {\arg \,\max _{s \in s\left( t \right){\ \mathrm{Neighbors}}}\overrightarrow {{\bf{s}}} \left( t \right) \cdot \overrightarrow {\bf{a}} \left( t \right)} \hfill & {\max \overrightarrow {\bf{a}} \left( t \right) > T_1} \hfill \end{array}} \right.$$

This allows the agent to search for new shapes but only on the meaning dimension in which attention is currently high—the attention is like blinders that keep other meaning categories from affecting the dynamics. We reasoned that the dynamics of this attention vector are such that attention increases according to the meaning of the current shape, and self-amplifies to lock the search and ignore nearby meaning peaks except for a single dimension of meaning. Thus, for the attention component at each dimension we have: **a**_i_(*t* + 1) = **a**_i_(*t*) + **a**_i_**s**_i_ – *γ*_a_**a**_i_ (here *γ*_a _is the reduction rate of attention).

This type of model yielded discovery of a single meaning category at a time (Fig. 4b)—for example, digit, digit, digit. However, unlike human players, the agents rarely escaped from this category and instead depleted all possible digit shapes. Thus, we have exploitation, but there is no exit from the exploitation phase.

To allow exit from the exploitation phase, we must include a second variable, that inhibits the attention vector once enough meaning has been collected. Previous work in the field of semantic search^36–39^ suggest that people switch between exploration and exploitation when the expected exploitation gain of the current category is equal to the expected gain of searching for new categories, i.e. people switch to exploration when the exploited category is depleted of words. In our paradigm, we find that players leave shape categories well before they are depleted^33^ (see Supplementary Fig. 3). Thus, creative search seems to transition from exploitation to exploration upon depletion in the novelty of shapes rather than the number of shapes remaining in a given category. We therefore term the parameter driving the end of exploitation as (novelty) Saturation (marked by the vector **b**_i_(*t*)).

When enough shapes from a category have been found, attention is shifted to new categories by the increase in the saturation variable. Saturation begins to rise when attention crosses a threshold *T*_2_. Saturation therefore has dynamics like **b**_i_ (*t* + 1) = **b**_i_ (*t*) + *Θ* (**a**_i_ – *T*_2_)**a**_i_**s**_i_ – *γ*_b_**b**_i_. Saturation then inhibits attention, for example: **a**_i_ (*t* + 1) = **a**_i_ (*t*) + **a**_i_**s**_i_/**b**_i_ – *γ*_a_**a**_i_. This process allows the searcher to return to exploration and discover new meaning dimensions (Fig. 4c).

We conclude that for a model to capture the behavioral observations it needs the following basic features: 1. Two internal variables, attention and saturation 2. Both attention and saturation accumulate with meaning. 3. Saturation inhibits attention (Fig. 4d).

While the specific model discussed so far resembles a feedforward loop architecture, the general framework of meaning-attention-saturation can be implemented by other possible models. In order to scan a wide space of possible models, we borrowed a technique from scanning of circuits in biological physics^13,40–44^. We constructed a framework with all possible signs of interactions between meaning, attention and saturation. We use the update rule (2). Attention rises with meaning and is inhibited by saturation. This class of models has two dimensionless parameters: the meaning accumulation rate *g* and the ratio of thresholds *α* = *T*_1_/*T*_2_ (see Fig. 4d):

3 $$\begin{array}{l}{\mathbf{a}}_{\mathrm{i}}\left( {t + 1} \right) = {\mathbf{a}}_{\mathrm{i}}\left( t \right) + g \left( {{\mathbf{a}}_{\mathrm{i}}^{w_1}{\mathbf{s}}_{\mathrm{i}}^{w_2}{\mathbf{b}}_{\mathrm{i}}^{w_3}\left( {\frac{{{\mathbf{a}}_{\mathrm{i}}}}{{\mathop {\sum }\nolimits {\mathbf{a}}_{\mathrm{i}}}}} \right)^{w_4} -\hskip 4pt {\mathbf{a}}_{\mathrm{i}}^{w_5}{\mathbf{s}}_{\mathrm{i}}^{w_6}{\mathbf{b}}_{\mathrm{i}}^{w_7}\left( {\frac{{{\mathbf{a}}_{\mathrm{i}}}}{{\mathop {\sum }\nolimits {\mathbf{a}}_{\mathrm{i}}}}} \right)^{w_8}} \right)\end{array}$$

4 $${\mathbf{b}}_{\mathrm{i}}\left( {t + 1} \right) =\ 	 {\mathbf{b}}_{\mathrm{i}}\left( t \right) + \Theta \left( {{\mathbf{a}}_{\mathrm{i}}-\alpha } \right){\mathbf{a}}_{\mathrm{i}}^{w_9}{\mathbf{s}}_{\mathrm{i}}^{w_{10}}{\mathbf{b}}_{\mathrm{i}}^{w_{11}} {\left({\frac{{{\mathbf{a}}_{\mathrm{i}}}}{{\mathop {\sum }\nolimits {\mathbf{a}}_{\mathrm{i}}}}} \right)^{w_{12}}} \\ 	- {\mathbf{a}}_{\mathrm{i}}^{w_{13}}{\mathbf{s}}_{\mathrm{i}}^{w_{14}}{\mathbf{b}}_{\mathrm{i}}^{w_{15}}{\left({\frac{{{\mathbf{a}}_{\mathrm{i}}}}{{\mathop {\sum }\nolimits {\mathbf{a}}_{\mathrm{i}}}}} \right)^{w_{16}}}$$

where *w*_i_ = 0, ± 1, indicates activation, inhibition or no interaction between the different components of the search. The term $\frac{{{\mathbf{a}}_{\mathrm{i}}}}{{\mathop {\sum }\nolimits {\mathbf{a}}_{\mathrm{i}}}}$ in Eq. (3) represents the relative attention spent in the specific meaning dimension i, as in neuronal models^45–48^. Each choice of a set of *w*_i_ is a specific realization of the general model, each such realization we term a circuit. In total, there are about 43 million different potential circuits. The number of circuits reduces to 6561 when we demand that meaning increases both saturation and attention, that saturation inhibits attention, and that saturation does not decay, so that searchers do not return to meaning dimensions already exploited (see Methods).

### FCD circuits capture creative search behavioral dynamics

We now invoke the scale invariance assumption for the search behavior. We scanned which of the 6561 circuits has FCD. A circuit is FCD if multiplying the meaning landscape scores by a scalar keeps the dynamics of the search unchanged. Our scan indicated that out of the 6561 circuits only 64 circuits show scale invariance. These circuits are all realizations of the FCD circuits known from cell-based circuits. In these FCD circuits, search is inherently robust to the absolute levels of the meaning scores, and senses only fold-changes of meaning. Below, we compare the 64 FCD circuits with 64 non-FCD circuits that demonstrate similar dynamics and have similar structure (see Supplementary Tables 1 and 2).

All circuits (both FCD and non-FCD) consist of two internal variables (attention and saturation), a fixed meaning landscape, and 2 model parameters (*g* and *α*). Therefore, the difference between circuits lies in the circuits’ topology (i.e., the connections between the different variables) while the number of variables and parameters is kept identical between different circuits.

To gather statistics on the dynamic behavior of the circuits, we simulated each circuit 25,000 times. For this purpose, we sampled 500 values of the parameters *g* and *α* log-uniformly from a wide range of values: *α*ϵ[1,1000], *g*ϵ[0.01,100]. For each pair of (*g*, *α*) we performed 500 runs with different random seeds (Methods).

First, we analyzed how robust is the exploration-exploitation behavior of each circuit by calculating the fraction of runs across (*g*, *α*)-parameter-space that show distinct periods of exploration and exploitation and cover at least 3 out of the 6 meaning dimensions of the simulated meaning landscape. FCD circuits showed higher probability for exploration-exploitation behavior, with all ten leading circuits being FCD (FCD median percentage of successful runs = 61%, 95% CI = [57%, 66%], non-FCD median percentage of successful runs = 50%, 95% CI = [44%, 58%], Mann–Whitney test *U* = 2377, *p* = 0.002, effect size = 0.33). This finding suggests that FCD circuits capture better the observed human exploration-exploitation behavior.

To further discern which circuit topology best describes the observed human behavior, we evaluated each circuit for the probability that its search dynamics produce exploration-exploitation durations that fall inside the convex hull of the experimental variation between different players’ exploration-exploitation durations (Fig. 4e). This probability measure indicates the ability of each circuit to capture the individual variations between human players by means of different model parameters.

We find that 6 of the 9 leading circuits are FCD (see Supplementary Table 3). The leading circuit is an FCD circuit with the following dynamics (Eqs. (3) and (4) with parameters— *w*_1_,*w*_6_,*w*_7_,*w*_8_,*w*_11_,*w*_12_ = 0,*w*_2_,*w*_4_,*w*_5_,*w*_9_,*w*_10_ = 1,*w*_3_ = −1):

5 $${\mathbf{a}}_{\mathrm{i}}\left( {t + 1} \right) = {\mathbf{a}}_{\mathrm{i}}\left( t \right) + g\left( \frac{{{\mathbf{s}}_{\mathrm{i}}}}{{{\mathbf{b}}_{\mathrm{i}}}}\frac{{{\mathbf{a}}_{\mathrm{i}}}}{\sum {\mathbf{a}}_{\mathrm{i}}} - {\mathbf{a}}_{\mathrm{i}} \right)$$

6 $${\mathbf{b}}_{\mathrm{i}}\left( {t + 1} \right) = {\mathbf{b}}_{\mathrm{i}}\left( t \right) + \Theta \left( {{\mathbf{a}}_{\mathrm{i}}-\alpha } \right){\mathbf{a}}_{\mathrm{i}}{\mathbf{s}}_{\mathrm{i}}$$

Its best counterpart out of the non-FCD circuits is given by (Eqs. (3) and (4) with parameters—*w*_1_,*w*_3_,*w*_5_,*w*_6_,*w*_8_,*w*_9_,*w*_11_,*w*_12_ = 0,*w*_2_,*w*_4_,*w*_7_,*w*_10_ = 1):

7 $${\mathbf{a}}_{\mathrm{i}}\left( {t + 1} \right) = {\mathbf{a}}_{\mathrm{i}}\left( t \right) + g\left( {{\mathbf{s}}_{\mathrm{i}}\frac{{{\mathbf{a}}_{\mathrm{i}}}}{{ {\sum {\mathbf{a}}_{\mathrm{i}}}}} - {\mathbf{b}}_{\mathrm{i}}} \right)$$

8 $${\mathbf{b}}_{\mathrm{i}}\left( {t + 1} \right) = {\mathbf{b}}_{\mathrm{i}}\left( t \right) + \Theta \left( {{\mathbf{a}}_{\mathrm{i}}-\alpha } \right){\mathbf{s}}_{\mathrm{i}}$$

We next preformed a model selection test for the different complexity incurred by the different topologies of the circuits. We used the Bayesian Information Criterion (BIC) to balance between model fidelity and complexity (similar results are found using the Akaike Information Criterion). We consider each interaction between variables in the circuit as a degree of freedom and compute the likelihood of each circuit by calculating the probability of the human behavioral data given the distribution created by simulations of the circuit. We find that among the ten leading circuits, seven circuits are FCD circuits and only three are non-FCD. The leading circuit is the same circuit presented in Eqs. (5) and (6) (see Supplementary Table 4).

The main difference between the two circuits above is that in the FCD circuit, different meaning-landscape scales show similar dynamics since a scalar can be gauged out from the circuit’s equations. This invariance to scale occurs because the saturation variable **b**_i_ normalizes out meaning **s**_i_ in Eq (5). In the non-FCD case, the system dynamics is affected strongly by multiplying the meaning landscape by a scalar in each meaning dimension. An analytical treatment of the leading FCD circuit dynamics is derived in Supplementary Note 4. Below we discuss simulation results of the model running on the meaning landscape.

### FCD circuits capture the exploration-exploitation correlation

**Fig. 5:**
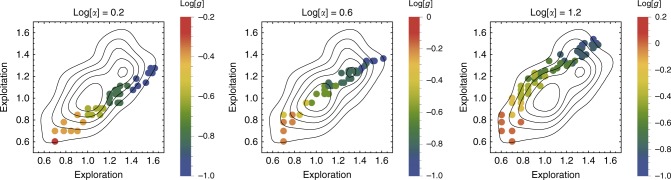
FCD model captures the variation between individuals in the durations of exploration-exploitation. In the FCD circuit (Eqs. (5) and (6)), a single parameter, *g*, the rate of meaning accumulation, sets the correlation between durations of exploration and exploitation. Shown are different simulation runs, grouped by different *α* values, while *g* values vary according to the color bar. Small *g* values show a thorough slow-to-discover/slow-to-drop behavior, while large *g* values show a mercurial quick-to-discover/quick-to-drop behavior, both as seen in the behavioral data. The second parameter, *α*, the attention threshold to saturation increase, controls the perpendicular principal component of behavioral data variation (see Supplementary Fig. 4, for similar simulation of the non-FCD circuit, Eqs. (7) and (8))

We analyzed the 25,000 simulations of the leading FCD circuit to see if it displays the correlation observed in players’ data between exploration and exploitation durations, which fall along a line-like continuum. We find that the variation between human players is captured by a single model parameter, *g*, the rate of meaning accumulation (see Fig. 5). The accumulation of meaning shortens the duration of the exploration phase and speeds the exit from a specific meaning dimension. Thus, different values of *g* allow modeling different ‘search personalities’ ranging from mercurial to thorough (Fig. 5). Similarly, the perpendicular axis of variation is primarily affected by the other parameter *α*, the normalized threshold for saturation to start accumulating (Fig. 5). We note that since FCD circuits are scale invariant, *g* is the only time-scale in the circuit, regardless of the average meaning score of different meaning dimensions, and hence naturally accounts for the correlation between exploration and exploitation phases.

### FCD dynamics are robust to variations in meaning perception

We next asked what might be the benefits of search dynamics governed by an FCD mechanism compared to a non-FCD mechanism? One possible answer is that FCD circuits provide search dynamics that is invariant to multiplying each different meaning dimension by a different scalar. We tested these predictions against the natural variations between meaning dimensions in the behavioral data and against larger variations induced in simulations.

**Fig. 6:**
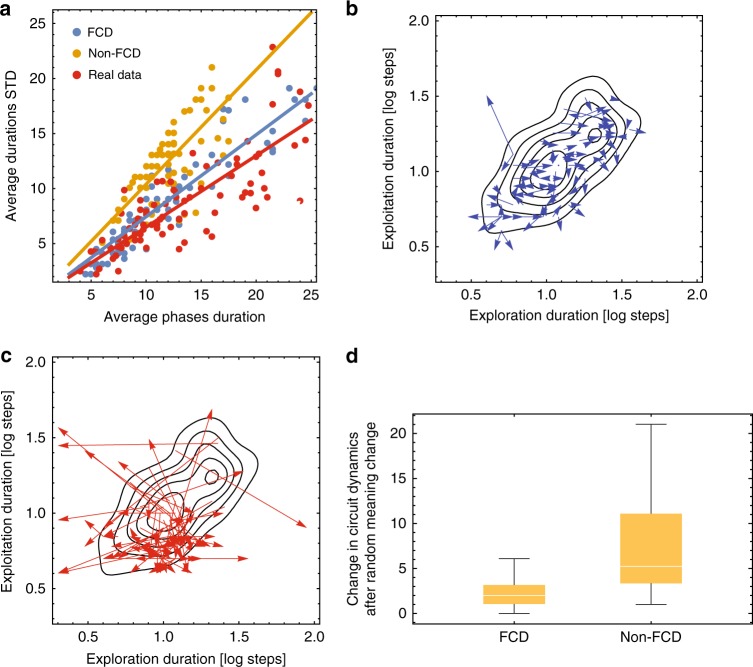
FCD dynamics are robust to variations in meaning perception. **a** The variance of the FCD circuit (blue) is significantly lower than that of the non-FCD circuit (orange), and aligns with the behavioral data (red) (FCD (blue): slope = 0.74, 95% CI = [0.71, 0.77], non-FCD (Orange): slope = 1.04, 95% CI = [1, 1.08], Behavioral data (red): slope = 0.65, 95% CI = [0.61, 0.69]). **b**, **c** FCD circuit dynamics are robust to large variations in meaning dimension’s average meaning score, while the non-FCD circuit dynamics change drastically. Arrows indicate the change in dynamics upon a random scaling of meaning dimensions for FCD response (blue arrows, **b**), and non-FCD response (red arrows, **c**). **d** Average arrow length in the case of **b** and **c**. FCD, median arrow norm = 2, 95% CI = [1.4, 2.2], non-FCD, median arrow norm = 5.2, 95% CI = [4.1, 6.3], Mann–Whitney Test, *U* = 1207, *p* < 10^−5^, effect size = 0.64. Center line in each box is the median value, box bounds are 25%, 75% quantiles, and whiskers show max and min values of data

A measure for the robustness of circuit dynamics to different meaning dimensions is the variance in exploration and exploitation durations (Methods). For both FCD and non-FCD circuits we find a strong correlation between phase duration and its variance (FCD, Spearman correlation *r* = 0.95, *p* < 10^−5^, non-FCD, Spearman correlation *r* = 0.84, *p* < 10^−5^). As predicted, the FCD circuit shows smaller variation compared with the non-FCD circuit (FCD, slope = 0.74, 95% CI = [0.71, 0.77], non-FCD, slope = 1.04, 95% CI = [1, 1.08], *p* < 0.001, Fig. 5a). Furthermore, the variation observed for the FCD circuit is close to the variation observed in the behavioral data (slope = 0.65, 95% CI = [0.61, 0.69], Fig. 6a).

We next compared the sensitivity of search dynamics to a global change in the meaning landscape for FCD and non-FCD circuits. The global change was introduced by multiplying each meaning dimension by a random scalar, sampled uniformly in log space between 10^−2 ^and 10^2^, thus creating large differences in the average meaning scores in different meaning dimensions (but maintaining their relative meaning within a category). For both FCD and non-FCD circuits we run for each parameter set of (*g*, *α*), 50 simulations of 200 moves each (Methods). We find that FCD search dynamics were robust to these global changes (Fig. 6b) whereas search dynamics of the non-FCD circuit changed drastically (Fig. 6c). To quantitate these changes, we calculated the change in median durations of exploration and exploitation. This change is the distance in the exploration-exploitation plane between the original point (simulation runs before the global change) and the shifted point after meaning rescaling. We find that the median distance in non-FCD is more than 2.5 times larger compared with FCD search (FCD, median arrow norm = 2, 95% CI = [1.4, 2.2], non-FCD, median arrow norm = 5.2, 95% CI = [4.1, 6.3], Mann–Whitney Test, *U* = 1207, *p* < 10^−5^, effect size = 0.64, Fig. 6d and Supplementary Fig. 5).

Notably, due to the large impact of the global meaning scaling on search dynamics in the non-FCD case, parameter sets initially providing search dynamics that lie in the convex hull of the human behavioral data are three times more likely to yield an exploration-exploitation relation that does not reside in the behavioral convex hull after the random scaling (FCD, %exit = 13%, 95% CI = [7%, 21%], non-FCD, %exit = 45%, 95% CI = [33%, 56%], see Fig. 6b, c).

The phenomenon of scale invariance has been previously linked with the concept of criticality. Evidence of critical-like neuronal activity was shown in in-vitro tissues recordings^49^, in vivo^50^, and fMRI data during rest and task performance^28,51,52^. These activities show the criticality hallmark of a 1/*f*^*β*^ power spectrum, indicating activity at many temporal and spatial scales^28,53,54^. Similar to FCD mechanisms, systems at criticality were shown to increase the system’s dynamic range^27,49,55^. Yet, there are important differences between the two scale invariant mechanism classes. First, a system at criticality inherently has many temporal scales. Therefore, it achieves a large dynamic range by virtue of all the temporal scales that already exist in the system. In contrast, an FCD circuit has one time scale and the circuit exactly adapts to the current scale of the input. Thus, a system at criticality would exhibit many time scales in its exploration-exploitation search dynamics, which is at odds with the well-defined exploration-exploitation time scale shown in FCD circuits and in our behavioral data (Supplementary Fig. 6).

A second difference between criticality and FCD is that a system at criticality with its long memory and correlations is more susceptible in its search dynamics to global scale changes and noise in parameters or inputs (see Supplementary Fig. 7). In contrast, an FCD circuit shows robust exploration-exploitation dynamics over a wide range of parameter sets and global scale changes.

## Discussion

Scale invariance plays an important role in cognition, where many processes exhibit similar behavior over several orders of magnitude^14–16,19^. Scale-invariant systems exhibit a large dynamic range, which allows them to respond accurately even if their inputs change drastically. However, there are few circuit-mechanisms that can explain scale invariance in behavior. Here, we show that creative search demonstrates scale invariance and suggest a specific mechanism, fold-change detection (FCD), as a governing design principle of creative search, narrowing down the set of possible circuits to a very few.

Our study shows that creative search can be modeled as a scale invariant search on a meaning landscape. This search mechanism is different from known search paradigms such as simulated annealing and naive Bayesian inference search^56^. It offers an effective temperature that rises with the recent success of the search (saturation variable) and a self-focusing mechanism (attention variable) that allows exploration of one specific meaning dimension at a time. Thus, the focus of the search shrinks and expands in exploitation and exploration phases in an organic way according to the progress of the search. This search process allows humans to achieve a difficult task—quickly finding meaningful solutions in a high-dimensional space of possibilities. Due to scale invariance, different players with different basal meaning attribution to shapes exhibit a common search pattern, consistent with our experimental results. A single parameter, the rate of meaning accumulation (*g*), explains the variation in players’ exploration-exploitation durations.

FCD in creative foraging predicts a search that is indifferent to the absolute scale of perceived meaning. Therefore, regardless of the content of the search, FCD ensures robust detection of the maxima of the sensed signal^5,13^. Thus, individual search strategies such as thorough explorations or mercurial discovery can be maintained despite differences in the basal meaning score of the meaning dimensions (e.g., search on the ubiquitous ‘digits’ dimension vs. a search on the scarcer ‘airplanes’ dimension). Lastly, FCD sensing allows comparing between different meaning dimensions on a ratiometric basis^7^. A ratiometric comparison for different categories was demonstrated in other cognitive abilities such as associating tones with crime severity^57^, weighing different types of rewards^30^, comparing performances at different domains (e.g., different sports and musical performances) and predicting future success from previous performance at other tasks (e.g., undergraduate GPA from 1st grade reading skills^57^).

We find that scale-invariant FCD circuits are more likely to show the observed exploration-exploitation behavior than non-FCD circuits. To understand why, we note that FCD circuit search dynamics depend only logarithmically on the parameters (*g*, *α*), thus maintaining a strong correlation between exploration and exploitation (see Figs. 5 and 6), whereas non-FCD circuits carry a larger change in response to changes in (*g*, *α*). Furthermore, since FCD search dynamics are robust to the global scale, the order of entry to different meaning dimensions does not change the exploration-exploitation dynamics, whereas in non-FCD circuits, the search can show a finite exploration-exploitation on one meaning dimension, but diverge on other meaning dimensions which differ in their basal meaning scale.

Recent studies have pointed to criticality as a possible mechanism for scale invariance in neuronal activity^49,50^. Here, we suggest a complementary class of mechanisms, FCD circuits, which provides scale invariant search dynamics. These two classes have different hallmarks that can be seen at the behavioral level. First, an FCD circuit carries a single well-defined time scale for search dynamics (enabling the variation along a mercurial/thorough search continuum due to the variation in this time scale between players). In contrast, criticality entails many time scales in activity, which predicts wide variability in exploration-exploitation dynamics within a single player (which are not observed in the human behavioral experiments). Second, due to its long correlations and memory, a system at criticality may respond more slowly to changes in the input compared with the fast response time of FCD circuits. The exact adaptation of FCD circuits drives its increased dynamic range, whereas systems at criticality achieve dynamic range by fluctuations at all scales of activity^27,49,55^. Lastly, while FCD search dynamics are robust to fluctuations in the meaning landscape, robust to global changes in meaning scaling, and robust to changes in the circuit’s parameters, a system at criticality may exhibit high sensitivity to noise in the inputs, changes of parameters and global changes in the input scale (but see also ref. ^58^).

The search for meaning through a network of shapes is akin to previous work on search in semantic networks^59–62^. These studies support Mednick’s theory of flat association curves^63^ by a computational analysis on networks of word associations^59^. Similarly, in our creative search model the parameter *g*, the accumulation rate of meaning, explains the variations in people’s association curves as expressed by their position on the exploration-exploitation correlation line. Thus, an interesting application of our results is testing whether in semantic search the exploration-exploitation times of different players are also correlated and span a continuum. A further test is whether semantic search dynamics are invariant across word categories, suggestive of an underlying FCD circuit.

An important question raised by our findings is which brain networks can implement an FCD circuit to support human creative search. A compelling answer may come from functional connectivity analysis that maps the relations between different brain networks during rest and task performance in creativity tasks^60,61,64^. In particular, Beaty and colleagues^60,61^ show that three main brain networks co-activate during a creativity task (Alternative Uses Test, AUT)—the default network, salience network, and executive control network. The functions of these networks seem to correspond to the three components of the proposed creative search model. The default network, involved in imagination, future-thinking, and assigning internal meaning^64,65^, can drive the values of shapes’ meaning. The salience network, involved in bottom-up attention to external and internal information and dynamic tuning between the default and executive control systems^66,67^ can drive the attention variable in creative search. Lastly, the executive control network, involved in evaluation of the creative process^68–70^, can drive the saturation variable. Notably, these co-activated networks antagonize each other in other cognitive tasks. This type of paradoxical activity, where the system’s components both drive and repress each other is akin to the incoherent FFL implementation of FCD^5,11–13^.

FCD may be also related to other systems in the brain. For example, studies of dopamine neurons in the reward system point to a sensing mechanism that perceives relative increase in rewards rather than absolute reward levels in static and dynamic comparisons^29–31^. Future studies can assess brain activity changes during the game itself, studying the correlation of activity in different brain regions during the game, in order to compare with the model’s predictions of attention and saturation variables, to attempt to locate brain dynamics that correspond to the dynamics of these variables.

The current model does not account for possible differences in the meaning accumulation rate for different meaning dimensions, nor for cultural and context dependent differences that may be critical to the understanding of individual creative search trajectories. Introducing a different meaning accumulation rate per meaning dimension can account for these modifications. Moreover, our current model is deterministic, ignoring noisy perception, and stochastic effects on our attention buildup and decision making. Future models can explore the effects of noise and stochasticity on creative foraging.

Taken together, our results suggest a scale invariant fold-change detection mechanism as a sensing mechanism for robust search that spans organisms from *E.coli* chemotaxis to human creative search.

## Methods

### Ethics statement

All experiments in this study adhere to the regulations and guidelines on the use of human subjects. The behavioral experimental protocol was approved by the Hebrew University IRB committee, and the brain activity experimental protocol was approved by the Weizmann Institute of Science ethics committee. All participants gave their informed consent to participate in the experiments.

### Meaning landscape construction

Every meaning category has a set of unique shapes that are not shared with other categories, these shapes are defined as core shapes. To construct the meaning landscape, we assign core shapes meaning scores in their specific dimension, by the amount of times that shape was chosen as a gallery shape by players. The more times a shape is chosen, the more meaning it has in that meaning dimension. Next, each core shape acts as a meaning source, spreading meaning to neighboring gallery shapes with an exponential decay of 10^−^^*d*^, where *d* is the minimal distance between the source shape and the receiving shape on the network of gallery shapes. Finally, each gallery shape acts as a source for all other shapes on the network of shapes with the same exponential decay of ${\mathrm{{10}}}^{- d_{\it{1}}}$, where *d*_1_ is the minimal distance between the source shape and the receiving shape on the network of all shapes up to three shapes away. We find that every shape is at most three steps away from a gallery shape.

### Pruning general model’s plausible circuits

There are 3^16^= 43,046,721 possible circuits in the general model. This number reduces to  177,147 under the assumptions that going back to a previous category is not allowed (hence *b*_i_ does not decay, removing the term ${\mathbf{a}}_{{\mathrm{i}}}^{w_{13}}{\mathbf{s}}_{{\mathrm{i}}}^{w_{14}}{\mathbf{b}}_{{\mathrm{i}}}^{w_{15}}\left(\frac{{{\mathbf{a}}_{{\mathrm{i}}}}}{{\mathop {\sum }\nolimits {\mathbf{a}}_{{\mathrm{i}}}}} \right)^{w_{16}}$), and that saturation (**b**_i_) dynamics should be activated by meaning, to make sure it represents meaning accumulation (hence *w*_10_ = 1). Preventing exponential growth of saturation by its own levels (setting *w*_11_ = 0), assuming saturation cannot increase attention levels (restricting (*w*_3_,*w*_7_) = (−1,0),(0,1),(−1,1)), and assuming that meaning must contribute to increasing attention levels (setting *w*_2_ = 1) results with a further reduction to 6561 circuits.

### The model search is robust to model parameters

In order to estimate parameter sensitivity, we scanned a range of 3–4 orders of magnitude in the model’s parameters. We randomly chose parameters from a log-uniformly distributed parameter range: *α*ϵ[1,1000], *g*ϵ[0.01,100]. We ran the model with 500 different parameter sets, each parameter set 500 times, with a cutoff of 500 steps per run. Each simulation produced a vector of meaning scores for each meaning dimension. We defined exploitation durations as the number of steps between the event of crossing the meaning threshold to the event of meaning score returning to threshold. Next, we defined exploitation durations as the number of steps between end of an exploitation phase to the start of the next one. A successful run is defined as a run where the simulated searcher finds at least three different meaning categories and that the exploitation phases do not overlap, i.e., there is a positive exploration phase duration. We find that for a large set of parameters, the models exhibit an exploration-exploitation behavior within the first 500 steps (see Supplementary Table 1). In particular, when measuring the median exploration and exploitation phase durations in the model and comparing it with the convex hull of players’ data, parameters range can vary over a 100-fold range: FCD model: *α*ϵ[1, 170], *g*ϵ[0.1, 2], non-FCD model: *α*ϵ[3, 856], *g*ϵ[1, 93].

### Calculation of phase duration-variance relation

We compared the variability in exploration and exploitation durations in the FCD and non-FCD circuits. For each parameter set (*g*, *α*) that is in the convex hull of the behavioral data (93 (*g*, *α*) pairs for the FCD circuit and 102 (*g*, *α*) pairs in the non-FCD circuit) we calculated the median and median deviation in exploitation duration and exploration duration across 500 simulations. We next plot the relation between the square root of the sum of variances ($\sqrt {\sigma _{\mathrm{R}}^2 + \sigma _{\mathrm{T}}^2}$) vs. the mean of the median durations ($\frac{1}{2}\left( {t_{\mathrm{R}} + t_{\mathrm{T}}} \right)$). For a similar result that computes the geometric mean of phases’ duration, see Supplementary Fig. 5).

### Simulations of large variations in averaged meaning score

We compared the FCD circuit and the non-FCD circuit response to large changes in the average score of each meaning dimension. For each parameter set (*g*, *α*) (93 FCD, 102 non-FCD) we created two types of simulations—First, we ran 50 simulations with the original meaning landscape and different random seeds (resulting in different search trajectories). Taking the median of exploration and exploitation durations over the 50 simulations defined the reference point in the exploration-exploitation plane. Next, we ran 50 simulations with the original meaning landscape rescaled by a random vector, which was sampled log-uniformly between [0.01, 100]. Taking the median of exploration and exploitation durations and computing the median response across the 50 simulations defined the shifted point in the exploration-exploitation plane. The distance between the shifted point and the reference point determine the scaling effect for that specific parameter set.

### Brain activity measurements

Participants: Seventeen healthy right handed subjects (ages 28 ± 3.8, 10 females) participated in the fMRI experiments. Fifteen of them participated in the full set-up of the experiment and their results are presented here. Fourteen filled a beauty, iconicity and weirdness evaluation questionnaire^35^ post the fMRI scan and their results are presented in Supplementary Fig. 2.

Stimuli: During the fMRI scan the created shapes were presented in blocks lasting 9s, followed by a 9s fixation screen. Each block consisted of nine images (1 s each): eight images in light green and one image in dark green. To reduce scan novelty effect, an extra block (which was not analyzed) was added to the beginning of each experiment, 29 blocks were presented in total. Each subject watched the same sequence of blocks. In the task, subjects were required to classify the stimuli according to color; light green (press 1) or dark green (press 2).

MRI data acquisition and preprocessing: The data were acquired on a 3 Tesla Trio Magnetom Siemens scanner at the Weizmann Institute of Science. Functional images of blood oxygenation level dependent (BOLD) contrast comprising of 46 axial slices were obtained with a T2*-weighted gradient echo planar imaging (EPI) sequence (3 × 3 × 3 mm voxel, TR= 3000 ms, TE= 30, flip angle = 75°, FOV 240 mm) covering the whole brain. Anatomical images for each subject were acquired in order to incorporate the functional data into the three-dimensional (3D) Talairach space using 3-D T1-weighted images with high-resolution (1 × 1 × 1 mm voxel, MPRAGE sequence, TR = 2300 ms, TE = 2.98 ms). The first 7-images of each functional scan (including the extra initial block and rest) were discarded. Functional scan preprocessing included 3D motion correction and filtering out of low frequency noise (slow drift), and spatial smoothing using an isotropic Gaussian kernel of 6 mm full-width-half-maximum (FWHM). The functional images were superimposed on 2D anatomic images and incorporated into the 3D data sets through trilinear interpolation. Statistical analysis was based on a general linear model in which all stimuli conditions were defined as predictors, and convolved with the hemodynamic response function (HRF).

Data analysis: To relate blocks' meaning to brain activity, a parametric GLM analysis was conducted. In this multi-subject, random effect analyses each block of shapes received a weight according to its meaning score, which was represented in the model as differential amplitude of the BOLD signal.

*T*-max correction analysis: To control for the Family-wise Error Rate (FWER), we repeated the parametric GLM analysis 1000 times with randomly permuted values of the meaning scores. We accumulated the *t*-values across the 1000 repeats and voxels to calculate the *t*-value distribution. We find that the *t*-value at *α* = 0.05 was *t*-value = 1.7, which is lower than the *t*-values of the parametric map (see Fig. 3).

### Fitting of decay functions to brain activity data

To find the best description of the meaning landscape, we calculated the correlations with brain activity data in the bilateral LOC region of human subjects (LOC was defined independently using an external localizing task^35^). We built three different versions of the meaning landscape, where each meaning landscape induces meaning to neighboring shapes in a different way. First, we used the same meaning landscape construction process as described above, where meaning decays by a constant factor with each step to a neighboring shape, *f*_1_(*λ*) = *λ*^*−d*^. Thus, shapes one move away decay by a factor of *λ*, shapes two moves away decay by a factor of *λ*^2^ and so on. We assigned for each shape in the brain imaging experiment its maximum meaning score across meaning dimensions, and then calculated the Spearman correlation and *p*-value for changing values of *λ*. We find that maximal correlation is achieved at $\lambda \simeq 10$ (see Fig. 6c). The maximal correlation plots are shown in Fig. 6d. We repeated the same construction process for a linear decay function *f*_2_(*λ*) = −*dλ*. We find that this decay function results in poor and non-significant correlations for positive meaning values.

### Code availability

Custom code for analysis of behavioral data and simulation of circuit topologies was written in Mathematica 11.3 (Wolfram) and can be found in the following link—https://github.com/uvhart/scaleinvariantcreativesearch. The analysis file contains: 1. Behavioral data analysis code, 2. FCD and Non-FCD circuit simulations code 3. fGn and fBm simulations and analysis code 4. Analysis of BIC/AIC scores for the FCD and non-FCD circuits compared to the behavioral data.

Brain data analysis was done using Brain Voyager QX 2.6 software package for brain imaging data analysis.

## Electronic supplementary material

Supplementary Information

Reporting Summary

## Data Availability

Behavioral data is available at https://github.com/uvhart/scaleinvariantcreativesearch. Brain activity data will be available from the authors upon request.
