# Publisher Correction: Creative exploration as a scale-invariant search on a meaning landscape

**DOI:** 10.1038/s41467-020-14901-0

**Published:** 2020-02-24

**Authors:** Yuval Hart, Hagar Goldberg, Ella Striem-Amit, Avraham E. Mayo, Lior Noy, Uri Alon

**Affiliations:** 1 Paulson School of Engineering and Applied Sciences, Harvard University, Cambridge, MA 02138 USA; 2 The Theatre Lab, Weizmann Institute of Science, 76100 Rehovot, Israel; 3 Department of Molecular Cell Biology, Weizmann Institute of Science, 76100 Rehovot, Israel; 4 Department of Neurobiology, Weizmann Institute of Science, 76100 Rehovot, Israel; 5 Department of Psychology, Harvard University, Cambridge, MA 02138 USA; 6 Department of Psychology, Ben Gurion University of the Negev, 8410501 Beer-Sheva, Israel

**Keywords:** Computational neuroscience, Scale invariance, Cognitive neuroscience, Systems biology

Correction to: *Nature Communications* 10.1038/s41467-018-07715-8; Article published online 21 December 2018

The original version of this Article incorrectly omitted the received/accepted dates of Received: 3 January 2018; Accepted: 7 November 2018. This has now been corrected in the PDF and HTML versions of the Article.

