# Supplementary material for: Creative exploration as a scale-invariant search on a meaning landscape
Source: Nat Commun. 2018 Dec 21;9:5411. doi: 10.1038/s41467-018-07715-8 (PMC6303308; doi:10.1038/s41467-018-07715-8)
Supplement: Supplementary file 1 — Supplementary Information [file 41467_2018_7715_MOESM1_ESM.pdf]

Creative exploration as a scale-invariant search on a meaning landscape – Supplementary Information

Hart et. al

Supplementary Note 1 The Creative Foraging Game induces a multi-dimensional meaning landscape

Our meaning landscape assigns meaning scores to a vector composed of 6 dimensions, each dimension is a visual meaning category players often find in their creative search. The 6 categories are constructed by clustering together shapes of exploitation phases of different players which share at least 2 shapes (see Ref[1]). This procedure creates 3 layers of description – First, the network of all shapes, connected by one move of a square between shapes. Second, the network of shapes chosen as 'beautiful and interesting', clustered by common phases of exploitation. Third, the meaning landscape, consisting of 6 meaning scores per shape.

For each meaning dimension we define core shapes as shapes which belong solely to a specific category (as opposed to shapes that belong to two different categories, and thus lie on the boundary between two categories). The meaning score of each core shape is the number of times different players chose this shape as 'interesting and beautiful'. Core shapes induce meaning to neighboring shapes in two stages. First, core shapes induce meaning to other chosen shapes on the network of chosen shapes (i.e. shapes players chose as 'interesting and beautiful', 2nd layer of description above). Second, chosen shapes induce meaning to all other shapes in the network of all shapes (1st layer of description above) distant at most 3 steps away. The meaning induced decays in both stages with distance as 10^{-d} where d is the distance between the two shapes (see Supplementary Figure 1 for a concrete example). This process repeats for all core shapes of every meaning dimension. The final score of each shape in each meaning dimension is the sum of all induced meaning scores from all core shapes (see Supplementary Figure 1). We verified that all shapes within the network of all shapes receive a meaning score in at least one meaning dimension.

Supplementary Note 2 Different decay functions produce lower correlations and p-values than exponential decay function of meaning induction

The meaning landscape portrays how meaning is induced from core shapes to all other shapes (see main text, Methods). The decay function of meaning to neighboring shapes dictates the form of the meaning landscape and hence search dynamics. We found a specific brain region, the lateral occipital complex (LOC) that dominantly responds to the meaning score of shapes. This activity correlated well with participants own evaluations of iconicity of the shape images (Supplementary Figure 2a), strengthening the validity of the meaning score induced by players game play. Thus, in order to explore which decay function describes best the meaning landscape, we correlate brain activity data (main text, Methods) with different meaning landscapes, generated by different decay functions and compare their correlation.

We analyzed three types of decay functions: the first is an exponential decay function which decreases meaning by a constant fold λ^{-d}, where d is the distance in moves between a shape and its source and λ is a fitting parameter. The second decay function is a linear decay function which decreases meaning by a constant amount, $-\lambda d$. The third is a power law decay function in

distance, $d^{-\lambda}$. For each decay function, we fitted λ to maximize the correlation of the meaning scores with the brain activity of participants. We then compared the resulting maximal correlation and p-value for each decay function.

Analysis of the exponential decay function shows high correlations for both left LOC and right LOC measured brain activity, with $\lambda = 10$ (Spearman correlation, lLOC: r=0.65, p-value=0.002, rLOC: r=0.46, p-value=0.01). We find that the linear decay function with one parameter is maximized when $\lambda = 0$, indicating a non-realistic scenario of no decay in meaning across shapes. Furthermore, the power-law decay function correlate poorly with participants' brain activity (Spearmen correlation, r= -0.01, p-value=0.63). This is explained by the slow decay of meaning near the source, which generates a more flattened meaning landscape.

We next compared the exponential decay function with linear and decay function of two parameters, such that the decay functions are $-\lambda d + \eta$, and $(\eta + d)^{-\lambda}$. Trying to adjust for the linear decay function results in negative values of the meaning score, again an unrealistic result. The results of the power-law fit for the optimal parameters ($\eta = 0.11, \lambda = 18$) are comparable to those of the exponential decay (Spearman correlation, left LOC: r=0.66, p-value=0.002, right LOC: r=0.48, p-value=0.009, see Supplementary Figure 2b,c), but at the cost of the addition of an extra parameter. Furthermore, the values of η, λ total to an effective decrease of 1/7 for the first move, similar to the decay in the case of the constant fold decrease of 1/10 found for the exponential decay function. We thus conclude that the exponential decay provides the best correlation with brain activity measurements.

Supplementary Note 3 Players leave exploitation before meaning is depleted

The mathematical model relies on a trade-off between attention and saturation - once a meaning dimension is chosen, the searcher employs a positive feedback loop to collect shapes with increased meaning, however, as shapes become more and more common, saturation increases also and the negative feedback loop makes the searcher leave the chosen meaning dimension before it is exhausted, at shapes with a high meaning gain. As such, the model predicts that searcher's trajectories in exploitation phases will not obey the law of diminishing returns of optimal foraging theory - shapes' meaning gain is higher towards the end of the cluster than at its beginning (Supplementary Figure 3a).

To test this prediction, we computed the meaning values of all players' shapes during exploitation phases and drew a curve of meaning vs. shapes' ordering for each exploitation phase. There are three possible shapes for the resulting curve: A linear curve of meaning over shapes ordering in the exploitation phase suggests a constant rate of meaning accumulation during exploitation. A convex curve suggests a monotonic decrease in the rate of meaning accumulation, corresponding to the 'law of diminishing returns'. We find that the average curve of an exploitation phase is concave (AUC = area under constant curve-area under behavioral curve=0.25, 95% CI=[0.22, 0.28]) and different from the constant rate of meaning accumulation (MW-test: t= 121230, $p<10^{-4}$, effect=0.16, Supplementary Figure 3b).

We validated the statistical significance of these results by randomly shuffling shapes' position in the exploitation phases and drawing curves of meaning vs. shapes' ordering. We find that random shuffling produces an average linear curve (AUC= -0.01, 95% CI=[-0.02,0.01])) which is significantly different from the convex curve when averaging over players data (MW-test: $p<10^{-4}$, effect=0.29, Supplementary Figure 3b, Inset). We find similar results for meaning vs. shape's timing, showing again a concave curve that does not obey the 'law of diminishing returns' in an even more pronounced way.

Supplementary Note 4 Analytical treatment of FCD circuit dynamics

In the main text we present an FCD circuit that shows the closest dynamics to human behavioral data. Here, we present an approximated analytical treatment of this circuit to extract the dependence of exploration and exploitation durations on the model's parameters.
We begin by considering the dynamic equations of the model -

$$(1)\ \mathbf{s}(t+1) = \begin{cases} Random\ neighbor & \vec{\mathbf{a}}(t) \le 1 \\ \arg\max_{s\in s(t)\ Neighbors} \vec{\mathbf{s}}\,\vec{\mathbf{a}}(t) & \vec{\mathbf{a}}(t) > 1 \end{cases}$$

$$(2)\ \frac{d\mathbf{a}_\mathrm{i}}{dt} = g\left(\frac{\mathbf{s}_\mathrm{i}}{\mathbf{b}_\mathrm{i}}\frac{\mathbf{a}_\mathrm{i}}{\sum \mathbf{a}_\mathrm{i}} - \mathbf{a}_\mathrm{i}\right)$$

$$(3)\ \frac{d\mathbf{b}_\mathrm{i}}{dt} = \Theta\left(\mathbf{a}_i - \alpha\right)\mathbf{a}_\mathrm{i}\mathbf{s}_\mathrm{i}$$

for clarity of the resulting behavior, we will treat a similar FCD description of the dynamics which considers only one search dimension and thus ignores the role of the normalization factor ($\sum \mathbf{a}_\mathrm{i}$). The dynamic equations are

$$(4)\ s(t+1) = \begin{cases} Random\ neighbor & a(t) \le 1 \\ \arg\max_{s\in s(t)\ Neighbors} s\, a(t) & a(t) > 1 \end{cases}$$

$$(5)\ \frac{da}{dt} = ga\left(\frac{s}{b} - 1\right)$$

$$(6)\ \frac{db}{dt} = \Theta\left(a - \alpha\right)a\, s$$

with initial conditions: $b(0) = b_0 = \langle s \rangle_{all\ shapes}, a(0) = a_0 \ll 1$.
There are 4 stages of interest, an exploration phase and 3 stages of the exploitation phase: In the first, attention and meaning score are low ($a(t) \ll 1, s \ll 1$), attention accumulates and the searcher performs a random walk. This is the exploration phase, and we can estimate its time by denoting $s = \langle s \rangle(1+\xi)$ where ξ is a random percent change in meaning at each step ($\xi \sim N(0,\sigma)$). To extract the exploration time, i.e. the time it takes a to cross 1 we note that $Log[a]$ is a purely random walk

$$(7)\ \frac{da}{dt} = ga\left(\frac{\langle s \rangle(1+\xi)}{\langle s \rangle} - 1\right) \Rightarrow \frac{dLog[a]}{dt} = g\xi$$

Hence, the mean exploration time is given by

$$(8)\ t_{explor} = \frac{1}{g\,\sigma^2}(Log[a_0^{-1}])^2$$

importantly, $t_{explor} \sim 1/g$.

In the next stage (beginning of exploitation phase), meaningful shapes are found and attention starts to accumulate but boredom does not rise yet ($1 < a(t) < \alpha, \frac{s}{b_0} > 1$). In this regime, we can approximate the equations by

(9) $\frac{da}{dt} = ga(\frac{s}{b_0} - 1)$

(10) $\frac{db}{dt} = 0$

which yields for the accumulation of attention

(11) $a(t) = \exp\left[g(\frac{s}{b_0} - 1)t\right] = \exp[g(F-1)t]$

Where F is the fold increase in meaning compared to its average level $b_0 = \langle s \rangle_{all\ shapes}$ (and we assumed F is roughly constant). This stage ends when $a(t) = \alpha$ so the time this stage takes is given by

(12) $t_{exploit,1} = \frac{1}{g(F-1)} Log[\alpha]$

The second stage of the exploitation phase is when both attention accumulates and boredom starts to accumulate as well ($a(t) > \alpha, \frac{s}{b_0} > 1$)

(13) $\frac{da}{dt} = ga\left(\frac{s}{b} - 1\right), a(0) = \alpha$

(14) $\frac{db}{dt} = a\, s, b(0) = b_0 \simeq 0$

To get a bound of the time scale of this time domain, we assume time scales separation, where attention is slowly affected by the still small values of boredom, so one has: $a(t) \simeq \exp[g(F-1)t]$ also in this domain. Then, boredom dynamics are given by

(15) $\frac{db}{dt} = \alpha \exp[g(F-1)t]\, s$

(16) $b(t) = \frac{\alpha\, s}{g(F-1)} \exp[g(F-1)t]$

Then, the dynamics of attention evolve until the rise in attention comes to a halt by the increase in boredom, this defines the second state time scale, $t_{exploit,2}$:

(17) $\frac{da}{dt} = ga\left(\frac{s}{b} - 1\right) = ga(\frac{g(F-1)}{\alpha} \exp\left[g(1-F)t_{exploit,2}\right] - 1)=0$

(18) $t_{exploit,2} = \frac{1}{g(F-1)} Log\left[\frac{g(F-1)}{\alpha}\right]$

At this stage, attention drops rapidly, as boredom levels increase exponentially fast to shut down attention. We assume boredom levels are much higher than shapes' meaning scores making attention dynamics decay exponentially with a characteristic time scale, $t_{exploit,3}$

(19) $\frac{da}{dt} = -ga$

(20) $t_{exploit,3} = \frac{1}{g}$

all together we have

(21) $t_{exploit} = \frac{1}{g}\left(\frac{1}{(F-1)} Log[\alpha] + \frac{1}{(F-1)} Log\left[\frac{g(F-1)}{\alpha}\right] + 1\right)$

Therefore, both time durations of exploration and exploitation depend on g, the rate of meaning accumulation and go as $1/g$. A scaling argument suggests that since g is the only time scale in the problem, while meaning scales are gauged out in FCD models, the only possible dependence of $t_{\text{exploit}}, t_{\text{explor}}$ is to scale like $1/g$. We further note that the dependency of exploration and exploitation phases on boredom threshold parameter is logarithmic.

Supplementary Note 5 Ranking circuits by their robustness properties

We ranked the circuits' viability to describe human behavior by 3 main parameters:
1. % runs that produce an exploration-exploitation behavior (see main text, Methods)
2. % runs that fall in the convex hull of human behavior (see main text, Fig 3e)
3. % coverage of the human convex hull by the simulated runs

For each of the 64 FCD circuits and the 64 non-FCD circuits we've compared these 3 scores by calculating the Pareto front of these circuits performance. A circuit is on the Pareto front if there does not exist a different circuit that can match or surpass its scores in all 3 parameters. We find that the Pareto front consists of 9 circuits, out of which 6 circuits are FCD and 3 are non-FCD. The list of circuits with their scores is detailed in Supplementary Table 3.

Supplementary Note 6 Balancing model complexity and model fidelity of FCD and non-FCD circuits

In this section, we describe a model selection procedure we preformed to account for the different complexity of the FCD and non-FCD circuits. We note that all the circuits examined in this work (FCD and non-FCD alike) are constructed with the same three variables (shapes meaning-attention-saturation), have only two parameters (g and α), and rely on the same meaning landscape. Since circuits ranking relies on their performance across different parameters' values, there is no risk for over-fitting the data.
However, when estimating circuits' performance, it is still interesting to account for their complexity as well as their fidelity. To do so, we counted the number of degrees of freedom for each circuit as the total number of interactions between the variables in each circuit (i.e. the number of non-zero values for the different w_{i}, see main text). We estimate the likelihood of each circuit by the following procedure –

1. We ran each circuit 500 times with different parameter values for (g,α), each run for 500 steps. Values for (g,α) were sampled log-uniformly (see Methods in the main text).
2. Each circuit shows a distribution of exploration-exploitation dynamics. We constructed a probability distribution on the exploration-exploration plane according to the simulations results.
3. We computed the likelihood of the behavioral data according to the circuit's probability distribution.

We used the Bayesian Information Criteria ($BIC = Ln(n) * DoF - 2Ln(Likelihood)$) which balances between the complexity of the model (the number of its degrees of freedom) and the fitting accuracy (the likelihood of the results). We find that 7 out of the 10 leading circuits are FCD circuits, with the top FCD circuit being the same FCD circuit chosen from the Pareto

analysis (see main text, Eqn 5-6 and Supplementary Table 4). Similar analysis using the Akaike Information criterion ($AIC = 2 * DoF - 2Ln(Likelihood)$) yields similar results (see Supplementary Table 4).

Supplementary Note 7 Non-FCD circuit dynamics capture the exploration-exploitation correlation

In the main text we presented how the leading FCD circuit captures the behavioral exploration-exploitation dynamics – both the global correlation between exploration duration and exploitation duration, and in how the two circuit's parameters, g and α, span the two main principal variation axes of the behavioral data (see Fig 5, main text).
Here, we present similar analysis for the leading non-FCD circuit. We find that the non-FCD circuit also shows correlation between exploration duration and exploitation duration (Spearman correlation, r=0.66, $p<10^{-5}$, see Supplementary Figure 4). This result is not surprising since the circuits are selected by their ability to cover as much of the convex hull of exploration-exploitation durations in the behavioral data. However, the parameters g and α show a weaker correspondence with the two main axes of variation in the behavioral data (Supplementary Figure 4 and compare with Fig 5 in the main text).

Supplementary Note 8 Scale changes of the meaning landscape incur dramatic changes in search dynamics only for the non-FCD circuit

In the main text, we examined the robustness of the FCD circuit and non-FCD circuit to both local and global scale changes of the meaning landscape. Local scale changes occur naturally between different categories within a game (Supplementary Figure 5a). Global scale changes were simulated by multiplying the entire meaning landscape by a fixed random number. We note that these global changes do not affect the form of the meaning landscape but just its overall scale. In the main text, we showed that while the FCD circuit buffers these changes, resulting in small changes to the search dynamics, the non-FCD circuit fluctuates greatly. Here, we present further analysis indicating the extent of these changes.

We start by considering local meaning changes effect on players' dynamics. We find that although there are 3-fold differences between the average meaning scores in extreme categories (Supplementary Figure 5a), their search dynamics patterns are similar (Supplementary Figure 5b). This finding suggests that players' search dynamics is the same regardless of the meaning category at play and its distribution of meaning scores, allowing players to search systematically whether the meaning score has low or high scores in that specific category and hence exhibiting scale invariance.

To further support this, we show that non-FCD circuits show higher variance and variance growth with respect to the mean of phases duration. Here, we calculated the dependency between the variance and the geometric mean of each run/game exploration-exploitation durations (compare with Fig 6a in the main text, which used the regular mean of phases durations). In Supplementary Figure 5c, we present the analysis of variance growth with geometric mean of the

two phases. While all variances grow monotonically with phases durations, both the FCD circuit and the real data curves show similar lower growth compared with the non-FCD circuit (FCD: slope=0.77, 95% CI=[0.73,0.8], non-FCD: slope=1.1, 95% CI=[1.05,1.15], Behavioral data: slope=0.67, 95% CI=[0.63,0.7]).

In Supplementary Figure 5d, we present the distribution of mean displacements of the search dynamics for the FCD vs. non-FCD circuits. We first calculate for each circuit and a choice of the simulation parameters (g,α), the mean durations of exploration and exploitation durations. Next, we perform the random scaling of the meaning landscape and calculate again for the same circuit with the same set of parameters the mean durations of exploration and exploitation phases. The Euclidian distance between the two points is the effect of rescaling on the search dynamics (see arrows in Fig 6b,c and the box whiskers plot on Fig 6d).

Supplementary Note 9 A system at criticality shows high variability in its exploration-exploitation dynamics

In the following we examine how known properties of systems at criticality would affect the creative search process. Since to the best of our knowledge, a concrete mechanism for creative search via a system in criticality does not exist, we instead analyze the implications of long temporal correlations which result in a power-law power spectrum through the analysis of two dynamic processes – fractional Brownian motion (fBm) process and its derivative, fractional Gaussian noise (fGn) process. The two dynamic processes are often used to model systems at criticality due to the long temporal memory they show. The processes are characterized by three parameters – μ, a drift parameter which biases the process towards positive or negative values, σ, a parameter that sets the variance level of the process, and H, termed the Hurst index which sets the raggedness of the process, and its self-similarity property: $y(at) = |a|^{-H}y(t)$. H is a real number between (0,1), with H>0.5 inducing positive correlation between increments of the process.

To quantify the effects of long temporal correlations on creative search dynamics, we assume that the attention variable in the search dynamics is driven by a fBm or fGn process, and measure the times at which attention levels cross a fixed threshold (set to 1). Each upward crossing event of the threshold initiates an exploitation phase, and each downward crossing of the threshold initiates an exploration phase. This simplified model for search dynamics of a system at criticality relieves the demand to find different meaning dimensions and focuses only on assessment of exploration-exploitation dynamics that are driven by a process with long temporal correlations.

For each process type, fBm and fGn, we simulated 500 different realizations of the process running for 300 time steps, with the following parameters: μ=0, σ=1, H=0.1 (fBm) or H=0.75 (fGn), and varying the threshold (0.5<th<2). We analyzed the variance in the mean duration of exploration-exploitation dynamics for different values of the mean duration of these phases and compare it with the same analysis done for the FCD and non-FCD circuits. To calculate the distribution of possible slopes we repeated each process 20 times and recorded the slope of the variance with mean exploration-exploitation phase duration. To create a distribution of slopes in

the FCD and non-FCD circuit behavior we used a bootstrapping method – we sampled the data (shown in Figure 6 in the main text) with replacements 1000 times and calculated the slope of the data for both FCD and non-FCD circuits.

We find that for both processes with long temporal correlation, the variance growth with the exploration-exploitation phase duration is higher compared with the behavioral data and with FCD circuits (Supplementary Figure 6a,b). fGn processes show variance growth (the slope of the lines at Supplementary Figure 6a) which is higher than the FCD circuits but comparable with the non-FCD dynamics, and fBm processes show extensive variance growth which exceeds both FCD and non-FCD circuits behavior (Supplementary Figure 6b). Finally, we performed the entire procedure while scanning different values of the Hurst index ($0.1<H<0.5$ for fBm, $0.5<H<0.9$ for fGn) and extracted the distribution of slope values for all processes. Hurst index in the fBm processes was limited to 0.5 due to a ceiling effect for higher exponents, where either exploration or exploitation phase duration reached the duration of the entire simulation (300 steps) or not converged at all, thus precluding variance assessment. We find that for different Hurst indices both fGn and (to a larger extent) fBm processes show higher variance growth compared with both the behavioral data and FCD circuits (see Supplementary Figure 6c,d).

We conclude that both fBm and fGn, two dynamic processes with long temporal correlations, show increased variance growth with the time of phase duration compared to FCD circuits and the behavioral data. This is a direct consequence of the many temporal scales that inherently exist in systems at criticality.

Supplementary Note 10 A system at criticality is sensitive to changes in its own parameters and to global changes in the scale of the meaning landscape

To assess the effects of a system in criticality on the exploration-exploitation phases, we assess the robustness of phases' duration upon changes to the system's parameters and to global changes in the scale of the meaning landscape. To that aim, we assume that the attention variable is driven by dynamic processes with long temporal correlations, as in the fBm and fGn processes.

For each process type, fBm and fGn, we simulated 500 different realizations of the process running for 300 time steps, with the drift term (μ) set to zero, and scanned various values of the variance parameter ($\sigma \in (0.5,2)$) and the Hurst index ($H \in (0.5,0.95)$). Scaling of the global meaning was done by multiplying the threshold (separating between exploration and exploitation behavior) by a random factor sampled log-uniformly between (0.01,100) as we did for the FCD and non-FCD circuits. We then calculated the distance between the exploration-exploitation (log) phase durations before and after the global change of scale. For each value of (σ, H) we averaged the distance measure across the 500 realizations.

We find that the two dynamic processes, fBm and fGn, show large deviations in their exploration-exploitation durations upon global scale changes, and also that they show high parameter sensitivity (Supplementary Figure 7a,b). While for the FCD circuit the median distance was about 2 (with 95% CI = [1.4, 2.2], see main text and Fig 6 therein), fBm processes yielded median distance of 26 (with 95% CI = [24,27], Supplementary Figure 7a) and large

variability in this measure for different values of (σ, H) (Standard deviation= ±7, see Supplementary Figure 7a, Inset). Similarly, fGn processes also yield large distances, albeit smaller than fBm processes, and large variation in their distance measure for different values of (σ, H) (median distance= 8, 95% CI = [7, 10], Standard deviation= 6, see Supplementary Figure 7b, and Supplementary Figure 7b, Inset).

We conclude that both fBm and fGn, two dynamic processes with long temporal correlations, show large deviations in their exploration-exploitation dynamics upon global scale changes and are sensitive to changes in their parameters. This is a result of the dynamics dependency on the critical system's parameters, and the many time scales present in systems at criticality.

We note that our scan considered two dynamic processes which are commonly used to model dynamic aspects of a critical system, and which are also common to models of critical brain activity. Further work is needed to assess whether globally these features persist for every system at criticality, and specifically to models of critical phenomena for creative search.

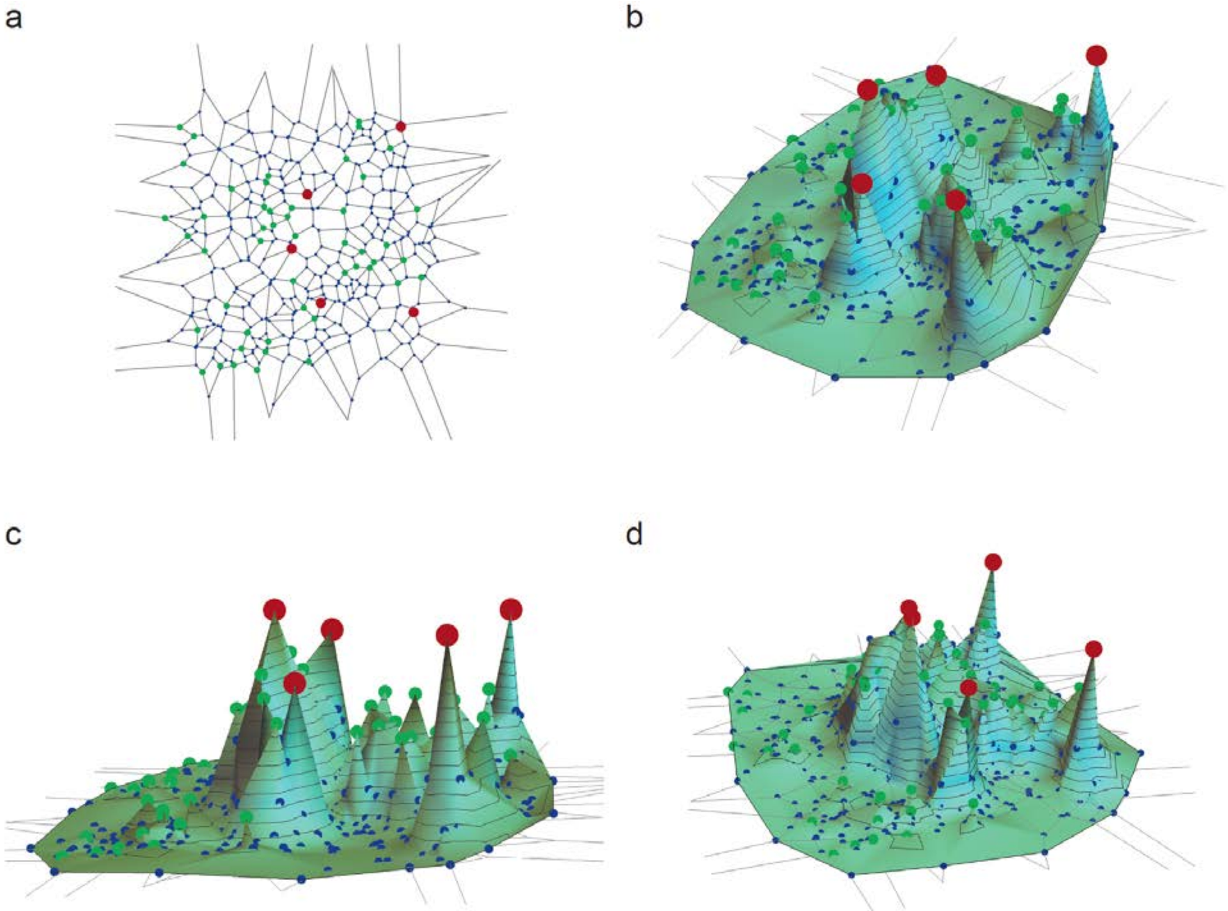

Supplementary Figure 1: Schematics of the meaning landscape. **a)** The network of shapes consists out of core shapes (marked in red), gallery shapes (marked in green) and all other shapes (marked in blue). Shapes are connected by one move of a square. **b-d)** Different views on the meaning landscape built on top of the network of shapes. At the first stage of building the meaning landscape, core shapes (red dots) are assigned a meaning score according to the number of times they were created by players in the game. Next, gallery shapes (green dots) receive meaning from core shapes that decay by 10^{-d}, where d is the distance between the two shapes. Then, all gallery shapes induce meaning to all shapes distant at most 3 moves away with the same decay function. This process repeats for all core shapes of every meaning dimension. The final score of each shape is the sum of all induced meaning scores from all core shapes. We verified that all shapes within the network receive a meaning score in at least one meaning dimension.

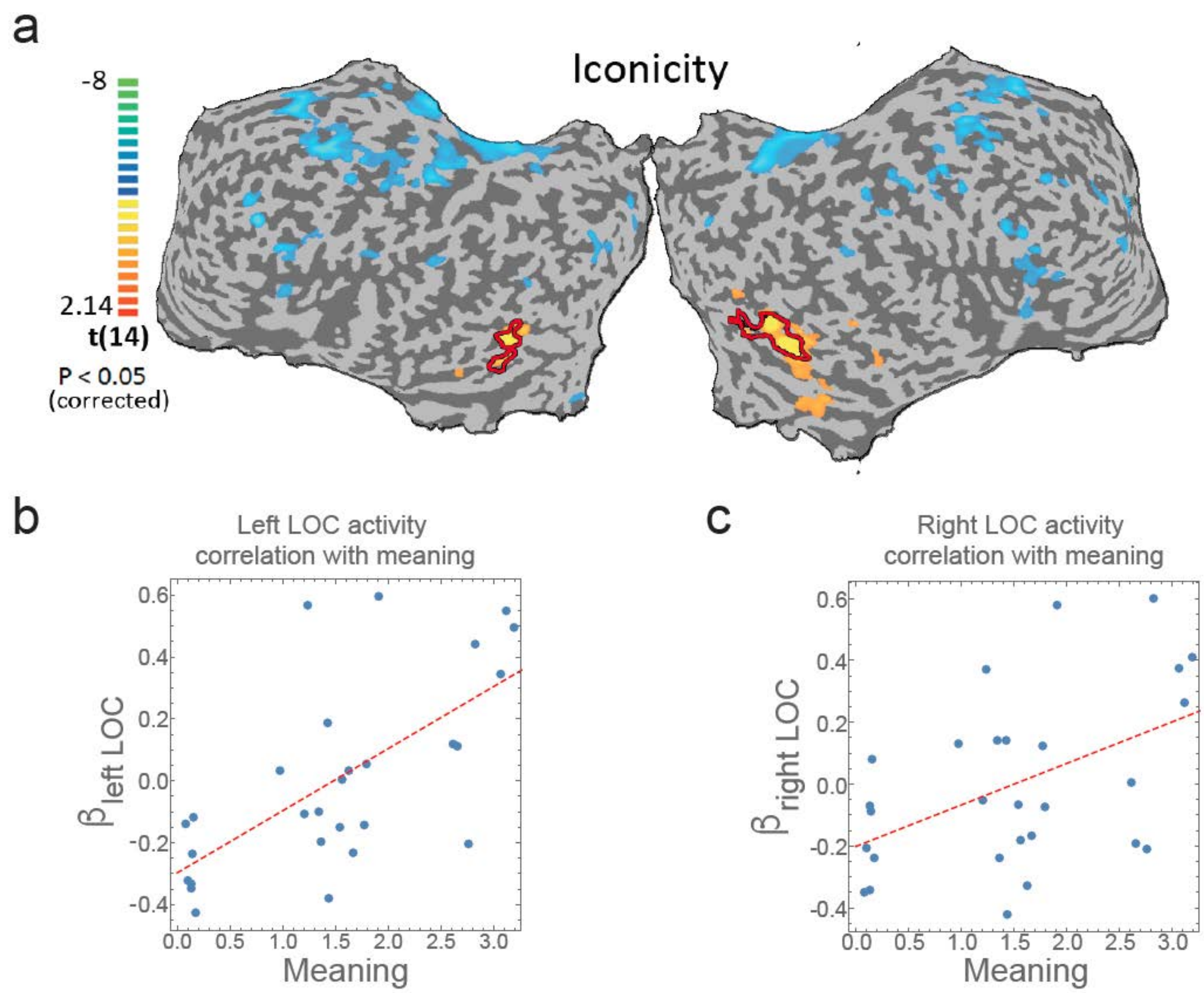

Supplementary Figure 2: Brain activity correlates with the meaning landscape. **a)** Parametric mapping of cortical activity with respect to participants' iconicity scores, presented on an unfolded cortex (N=14, corrected for multiple comparisons, $p<0.05$). Color scale indicates t values. Yellow-orange scale represents regions which showed positive parametric relation with iconicity scores. Blue-green scale represents regions which showed negative parametric relation with iconicity scores (adapted from Goldberg et al[2] and compare with Fig 3a in the main text). **b+c)** Correlations of LOC activity and meaning scores created by the power-law decay function ($(\eta + d)^{-\lambda}$). Scatter plots present the relation between the averaged meaning of blocks (x axis) and the averaged brain activity (N=14, normalized beta weight, y axis) in bilateral LOC. Each dot represents one block. Spearman correlation; left LOC: r=0.66, p-value=0.002, right LOC: r=0.48, p-value=0.009.

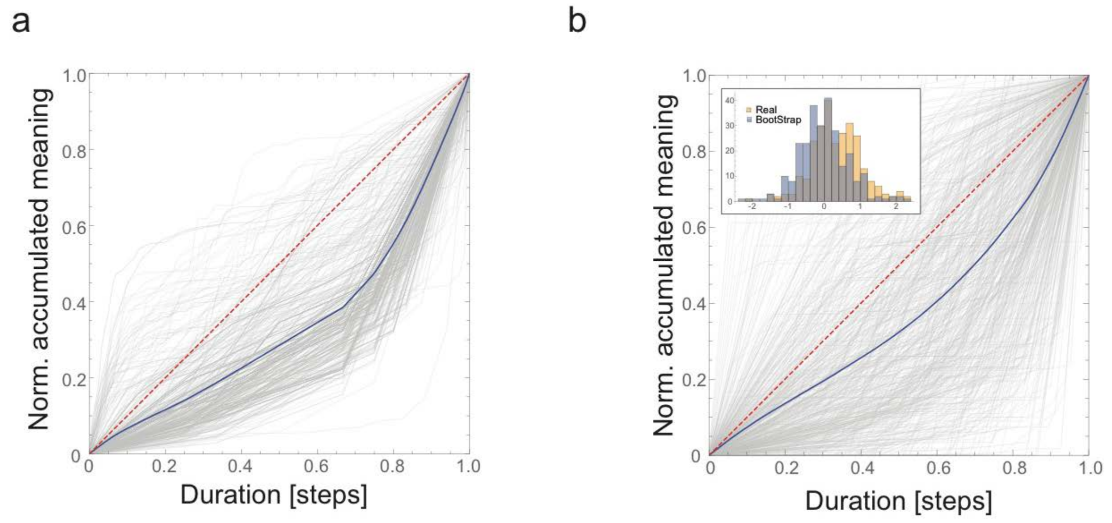

Supplementary Figure 3: Exploitation phases show "no diminishing returns" in the model and in the behavioral data. a) Model simulations (see main text, eqn. (5-6)) on the meaning landscape show "no diminishing returns" in the exploitation phase. Phases of exploitation durations and the corresponding accumulation of meaning scores of the chosen shapes were rescaled between 0-1 to compare between different phases. Grey lines are individual clusters of shapes found in exploitation phases. Blue solid line, average over all individual curves. Red line shows constant accumulation of meaning, and separates between curves of "diminishing returns" (above the line) and curves of "no diminishing returns" (below the line). **b)** Players' behavior shows "no diminishing returns". Grey lines are individual clusters of shapes found in exploitation phases. Blue solid line, average over all individual curves. Red line, the separator between curves of "diminishing returns" (above the line) and curves of "no diminishing returns" (below the line). **Inset,** the distribution of players' accumulation of meaning curves (as reflected by their AUC= area under the constant accumulation of meaning curve- area under the curve) is significantly different ($p<10^{-4}$) from a bootstrapped version where gallery-shapes locations were randomized (and thus shows on average a constant accumulation of meaning, and an AUC value of 0).

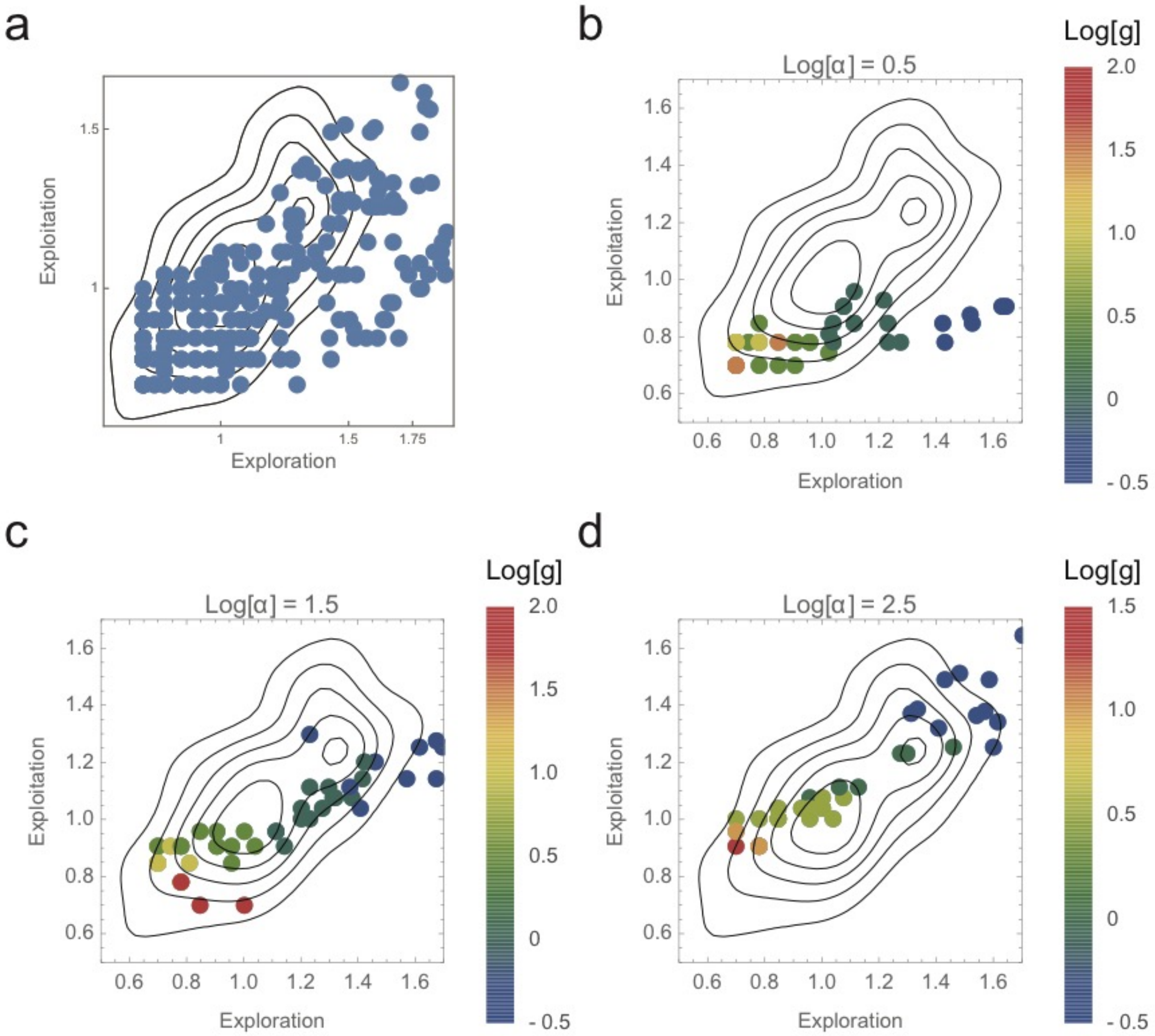

Supplementary Figure 4: Non-FCD circuit dynamics capture the exploration-exploitation global correlation but show weaker correspondence with the two axes of variation in the behavioral data. **a)** The results of search dynamics for the leading non-FCD circuit in coverage of the behavioral data's search dynamics convex hull. The correlation of points is similar to that of the behavioral data (Spearman correlation, r=0.66 , $p<10^{-5}$, compare with Fig 4 in the main text). **b,c,d)** The dependency of the non-FCD circuit's search dynamics on changes in the parameter g, the rate of meaning accumulation, for different fixed values of the parameter α, the attention threshold to increase saturation. The two parameters, g and α, show a weaker correspondence to the two main axes of variation in the behavioral data. Compare with Fig 5 in the main text.

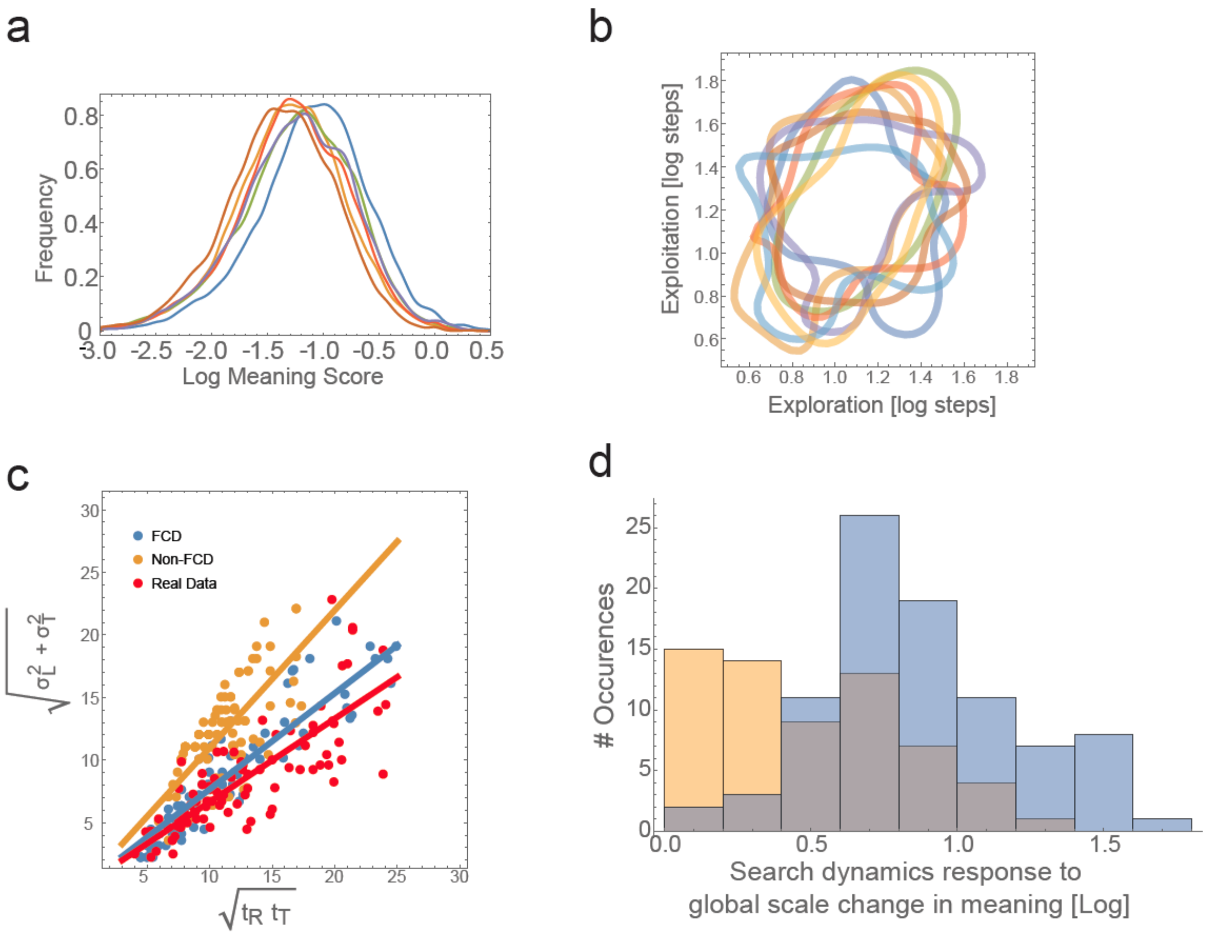

Supplementary Figure 5: Scale changes of the meaning landscape incur dramatic changes in search dynamics only for the non-FCD circuit. **a)** Different meaning categories carry different meaning scales, shown are histograms of shapes' meaning scores per meaning categories. Categories exhibit Log-Normal distribution of meaning scores, with the highest meaning category showing ~3 times higher meaning scores compared to the lowest meaning scores category (blue line compared with the red line). **b)** The search dynamics of different meaning categories cover a similar region in the exploration-exploitation duration plane. This suggests the search dynamics are invariant under scale transformations. **c)** The variance of FCD circuit is significantly lower than the variance of the non-FCD circuit, and aligns with the behavioral data. x-axis is the geometric mean of step durations in exploration and exploitation phases, y-axis is the averaged standard variation of these two phases. FCD (blue): slope=0.77, 95% CI=[0.73,0.8], non-FCD (Orange): slope=1.1, 95% CI=[1.05,1.15], Behavioral data (Red): slope=0.67, 95% CI=[0.63,0.7]. **d)** Distributions of FCD (orange) and Non-FCD responses to global changes of the meaning scale. We calculated the mean point in exploration-exploitation duration plane for each circuit type for different parameter sets. We then repeated this calculation with multiplying the entire meaning landscape by a random scalar. The Euclidean distance between the two points (original and after scaling) in the exploration-exploitation duration plane is considered as the circuit's response to scaling.

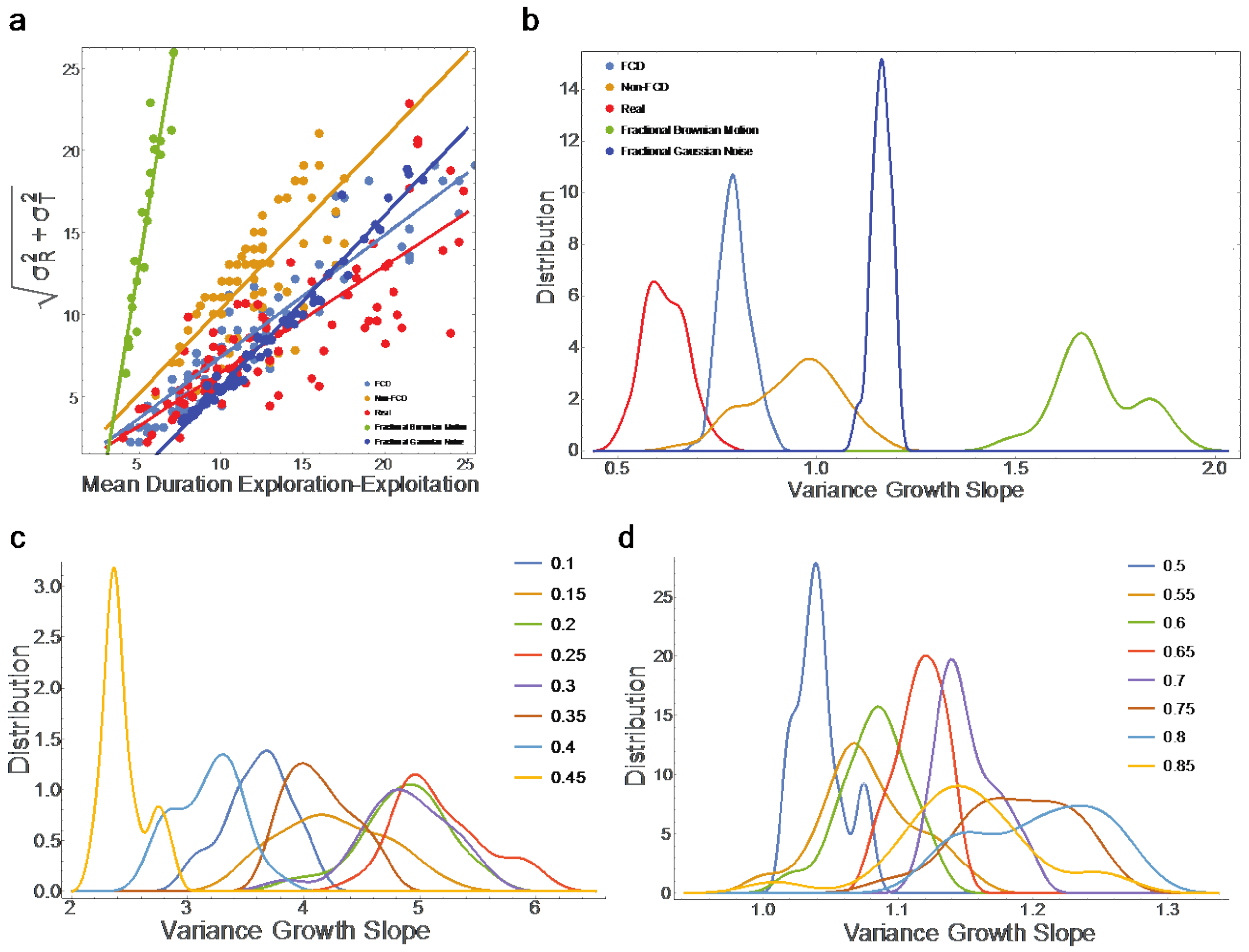

Supplementary Figure 6: Systems at criticality show higher variability in their exploration-exploitation dynamics. a) The combined standard deviation of exploration and exploration search dynamics vs. the average duration of the two phases. Plotted are dots for data points and solid lines for the best linear fit in the region shown. Presented are behavioral data (red), FCD circuit (light blue), non-FCD (orange), fractional Gaussian noise process (dark blue), and fractional Brownian Motion (green). **b)** The distributions of the slopes of the standard deviation vs. phase duration for the different models. The distribution for the behavioral data, FCD, and non-FCD was calculated by random sampling of the dots with replacements and calculating the slopes. The distribution for the fGn and fbm processes was calculated by repeating the random process with several thresholds (see Supplementary Note 9 for more details). The slopes distributions indicate that fractional Gaussian noise and to a far larger extent fractional Brownian motion show higher variability growth. Shown are behavioral data (red), FCD circuit (light blue), non-FCD (orange), fractional Gaussian noise process (dark blue), and fractional Brownian Motion (green). Scan parameter values: μ=0, σ=1, H=0.1 (fBm) and H=0.75 (fGn), 0.5<th<2. **c,d)** A scan of the variance slope for different Hurst index values ($0.1 \leq H \leq 0.45$ for fBm, $0.5 \leq H \leq 0.85$, see legend of each panel) shows higher variance growth for both fBm (panel c) and fGn (panel d) dynamic processes. All other parameter values were as in **b)**.

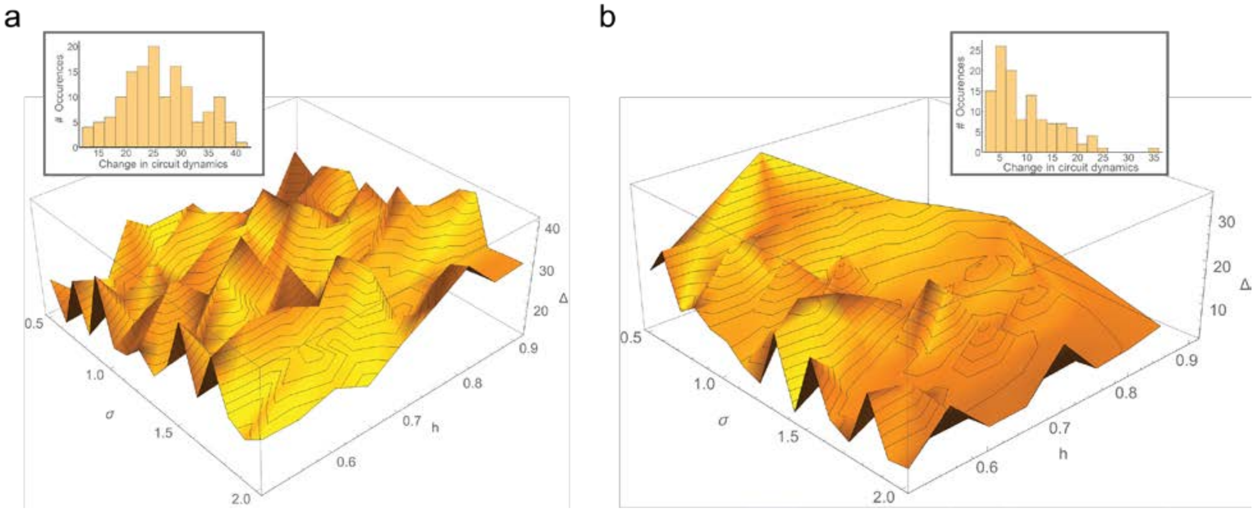

Supplementary Figure 7: A system at criticality is sensitive to changes in its own parameters and to global changes in the scale of the meaning landscape. a) fBm processes show high deviations in their exploration-exploitation dynamics upon global scale changes (median distance=26, 95% CI = [24,27]). Distances vary greatly upon changes in the systems' two main parameters, its variance and Hurst index (σ, H) (Standard deviation= ±7). **b)** fGn processes show large deviations in their exploration-exploitation dynamics upon global scale changes and upon changes to the system's parameters, (σ, H) (median distance= 8, 95% CI = [7, 10], Standard deviation= 6).

Supplementary Table 1: List of FCD circuits – model equations and matching scores to behavioral data

	Model	# successful Runs	# runs in HM Convex Hull	Vol(HM⋂M)
1	$a' == \left(\frac{a}{\Sigma a}\frac{s}{b} - a\right) g \quad b' == a\, s\, \theta[a - \alpha]$	548[69%]	91[11%]	0.527
2	$a' == \left(-1 + \frac{a}{\Sigma a}\frac{s}{b}\right) g \quad b' == a\, s\, \theta[a - \alpha]$	461[92%]	102[20%]	0.524
3	$a' == \left(\frac{a}{\Sigma a}\frac{s}{b} - a\right) g \quad b' == s\, \theta[a - \alpha]$	664[70%]	124[13%]	0.481
4	$a' == \left(-a + \frac{s}{b} a\right) g \quad b' == a\, s\, \theta[a - \alpha]$	470[94%]	77[15%]	0.463
5	$a' == \left(\frac{s}{b} - \frac{a}{\Sigma a} a\right) g \quad b' == \frac{a}{\Sigma a} s\, \theta[a - \alpha]$	231[46%]	30[6%]	0.46
6	$a' == \left(\frac{a}{\Sigma a}\frac{s}{b} - \frac{a}{\Sigma a} a\right) g \quad b' == \frac{a}{\Sigma a} s\, \theta[a - \alpha]$	419[56%]	34[5%]	0.451
7	$a' == \left(\frac{s}{b} - a\right) g \quad b' == \frac{a}{\Sigma a} a\, s\, \theta[a - \alpha]$	954[64%]	48[3%]	0.45
8	$a' == \left(\frac{a}{\Sigma a}\frac{s}{b} - a\right) g \quad b' == \frac{a}{\Sigma a} s\, \theta[a - \alpha]$	344[69%]	60[12%]	0.443
9	$a' == \left(-1 + \frac{a}{\Sigma a}\frac{s}{b}\right) g \quad b' == \frac{a}{\Sigma a} a\, s\, \theta[a - \alpha]$	456[91%]	101[20%]	0.44
10	$a' == \left(\frac{a}{\Sigma a}\frac{s}{b} - a\right) g \quad b' == \frac{a}{\Sigma a} a\, s\, \theta[a - \alpha]$	362[72%]	50[10%]	0.414
11	$a' == \left(-1 + \frac{a}{\Sigma a}\frac{s}{b}\right) g \quad b' == s\, \theta[a - \alpha]$	467[93%]	122[24%]	0.4
12	$a' == \left(\frac{s}{b} - a\right) g \quad b' == s\, \theta[a - \alpha]$	317[63%]	20[4%]	0.373
13	$a' == \left(-a + \frac{s}{b} a\right) g \quad b' == \frac{a}{\Sigma a} s\, \theta[a - \alpha]$	804[96%]	157[19%]	0.37
14	$a' == \left(-1 + \frac{a}{\Sigma a}\frac{s}{b}\right) g \quad b' == \frac{a}{\Sigma a} s\, \theta[a - \alpha]$	464[93%]	152[30%]	0.367
15	$a' == \left(-a + \frac{s}{b} a\right) g \quad b' == s\, \theta[a - \alpha]$	469[94%]	85[17%]	0.366
16	$a' == \left(\frac{a}{\Sigma a}\frac{s}{b} - \frac{a}{\Sigma a} a\right) g \quad b' == s\, \theta[a - \alpha]$	425[58%]	29[4%]	0.362
17	$a' == \left(\frac{s}{b} - a\right) g \quad b' == a\, s\, \theta[a - \alpha]$	294[59%]	14[3%]	0.36
18	$a' == \left(-1 + \frac{s}{b}\right) g \quad b' == s\, \theta[a - \alpha]$	234[47%]	80[16%]	0.359
19	$a' == \left(\frac{s}{b} - a\right) g \quad b' == \frac{a}{\Sigma a} s\, \theta[a - \alpha]$	329[66%]	27[5%]	0.359
20	$a' == \left(\frac{s}{b} - \frac{a}{\Sigma a} a\right) g \quad b' == s\, \theta[a - \alpha]$	228[46%]	18[4%]	0.319
21	$a' == \left(-a + \frac{s}{b} a\right) g \quad b' == \frac{a}{\Sigma a} a\, s\, \theta[a - \alpha]$	852[96%]	149[17%]	0.314
22	$a' == \left(-\frac{a}{\Sigma a} a + \frac{a}{\Sigma a}\frac{s}{b} a\right) g \quad b' == \frac{a}{\Sigma a} s\, \theta[a - \alpha]$	373[90%]	63[15%]	0.312
23	$a' == \left(-\frac{a}{\Sigma a} + \frac{a}{\Sigma a}\frac{s}{b}\right) g \quad b' == s\, \theta[a - \alpha]$	209[42%]	25[5%]	0.303
24	$a' == \left(-\frac{a}{\Sigma a} a + \frac{a}{\Sigma a}\frac{s}{b} a\right) g \quad b' == s\, \theta[a - \alpha]$	436[87%]	64[13%]	0.295
25	$a' == \left(\frac{s}{b} - \frac{a}{\Sigma a} a\right) g \quad b' == \frac{a}{\Sigma a} a\, s\, \theta[a - \alpha]$	222[44%]	16[3%]	0.292
26	$a' == \left(\frac{a}{\Sigma a}\frac{s}{b} - \frac{a}{\Sigma a} a\right) g \quad b' == \frac{a}{\Sigma a} a\, s\, \theta[a - \alpha]$	277[55%]	11[2%]	0.285
27	$a' == \left(\frac{a}{\Sigma a}\frac{s}{b} - \frac{a}{\Sigma a} a\right) g \quad b' == a\, s\, \theta[a - \alpha]$	253[51%]	14[3%]	0.25
28	$a' == \left(\frac{s}{b} - \frac{a}{\Sigma a} a\right) g \quad b' == a\, s\, \theta[a - \alpha]$	192[38%]	7[1%]	0.21
29	$a' == \left(-a + \frac{a}{\Sigma a}\frac{s}{b} a\right) g \quad b' == \frac{a}{\Sigma a} s\, \theta[a - \alpha]$	397[79%]	16[3%]	0.198
30	$a' == \left(-1 + \frac{s}{b}\right) g \quad b' == \frac{a}{\Sigma a} a\, s\, \theta[a - \alpha]$	114[23%]	25[5%]	0.194
31	$a' == \left(-a + \frac{a}{\Sigma a}\frac{s}{b} a\right) g \quad b' == s\, \theta[a - \alpha]$	399[80%]	19[4%]	0.19
32	$a' == \left(-\frac{a}{\Sigma a} + \frac{a}{\Sigma a}\frac{s}{b}\right) g \quad b' == \frac{a}{\Sigma a} a\, s\, \theta[a - \alpha]$	290[40%]	20[3%]	0.187
33	$a' == \left(-a + \frac{a}{\Sigma a}\frac{s}{b} a\right) g \quad b' == a\, s\, \theta[a - \alpha]$	386[77%]	6[1%]	0.162
34	$a' == \left(-\frac{a}{\Sigma a} a + \frac{s}{b} a\right) g \quad b' == s\, \theta[a - \alpha]$	436[48%]	61[7%]	0.12
35	$a' == \left(-\frac{a}{\Sigma a} + \frac{a}{\Sigma a}\frac{s}{b}\right) g \quad b' == a\, s\, \theta[a - \alpha]$	188[38%]	4[1%]	0.117
36	$a' == \left(-1 + \frac{s}{b}\right) g \quad b' == a\, s\, \theta[a - \alpha]$	50[10%]	4[1%]	0.105
37	$a' == \left(-\frac{a}{\Sigma a} a + \frac{s}{b} a\right) g \quad b' == \frac{a}{\Sigma a} s\, \theta[a - \alpha]$	421[49%]	203[24%]	0.092
38	$a' == \left(-a + \frac{a}{\Sigma a}\frac{s}{b} a\right) g \quad b' == \frac{a}{\Sigma a} a\, s\, \theta[a - \alpha]$	402[80%]	5[1%]	0.062
39	$a' == \left(-\frac{a}{\Sigma a} a + \frac{a}{\Sigma a}\frac{s}{b} a\right) g \quad b' == a\, s\, \theta[a - \alpha]$	438[88%]	13[3%]	0.039
40	$a' == \left(-\frac{a}{\Sigma a} + \frac{a}{\Sigma a}\frac{s}{b}\right) g \quad b' == \frac{a}{\Sigma a} s\, \theta[a - \alpha]$	304[30%]	5[0%]	0.029
41	$a' == \left(-1 + \frac{s}{b}\right) g \quad b' == \frac{a}{\Sigma a} s\, \theta[a - \alpha]$	72[14%]	11[2%]	0.024
42	$a' == \left(-\frac{a}{\Sigma a} a + \frac{s}{b} a\right) g \quad b' == a\, s\, \theta[a - \alpha]$	482[60%]	7[1%]	0.007
43	$a' == \left(-\frac{a}{\Sigma a} a + \frac{a}{\Sigma a}\frac{s}{b} a\right) g \quad b' == \frac{a}{\Sigma a} a\, s\, \theta[a - \alpha]$	437[87%]	7[1%]	0.007

Supplementary Table 2: List of non-FCD circuits – model equations and matching scores to behavioral data

	Model	⌗ successful Runs	⌗ runs in HM Convex Hull	Vol(HM⋂M)
1	$a' == \left(\frac{a}{\Sigma a} s - \frac{a}{\Sigma a} a b\right) g \quad b' == a s \theta[a-\alpha]$	227[45%]	36[7%]	0.503
2	$a' == \left(\frac{a}{\Sigma a} s - b\right) g \quad b' == s \theta[a-\alpha]$	359[72%]	103[21%]	0.502
3	$a' == \left(s - \frac{a}{\Sigma a} a b\right) g \quad b' == \frac{a}{\Sigma a} a s \theta[a-\alpha]$	203[41%]	24[5%]	0.5
4	$a' == \left(s - b\right) g \quad b' == s \theta[a-\alpha]$	216[43%]	37[7%]	0.498
5	$a' == \left(\frac{a}{\Sigma a} s - b\right) g \quad b' == a s \theta[a-\alpha]$	357[71%]	47[9%]	0.495
6	$a' == \left(s - \frac{a}{\Sigma a} a b\right) g \quad b' == a s \theta[a-\alpha]$	189[38%]	28[6%]	0.479
7	$a' == \left(\frac{a}{\Sigma a} s - a b\right) g \quad b' == s \theta[a-\alpha]$	242[48%]	29[6%]	0.473
8	$a' == \left(\frac{a}{\Sigma a} s - b\right) g \quad b' == \frac{a}{\Sigma a} s \theta[a-\alpha]$	362[72%]	90[18%]	0.472
9	$a' == \left(\frac{a}{\Sigma a} s - \frac{a}{\Sigma a} b\right) g \quad b' == a s \theta[a-\alpha]$	331[66%]	38[8%]	0.466
10	$a' == \left(s - a b\right) g \quad b' == \frac{a}{\Sigma a} s \theta[a-\alpha]$	247[50%]	25[5%]	0.461
11	$a' == \left(\frac{a}{\Sigma a} s - a b\right) g \quad b' == a s \theta[a-\alpha]$	239[48%]	40[8%]	0.449
12	$a' == \left(\frac{a}{\Sigma a} s - b\right) g \quad b' == \frac{a}{\Sigma a} a s \theta[a-\alpha]$	362[72%]	58[12%]	0.448
13	$a' == \left(\frac{a}{\Sigma a} s - a b\right) g \quad b' == \frac{a}{\Sigma a} a s \theta[a-\alpha]$	250[50%]	32[6%]	0.438
14	$a' == \left(\frac{a}{\Sigma a} s - \frac{a}{\Sigma a} a b\right) g \quad b' == \frac{a}{\Sigma a} a s \theta[a-\alpha]$	214[43%]	30[6%]	0.427
15	$a' == \left(\frac{a}{\Sigma a} s - \frac{a}{\Sigma a} b\right) g \quad b' == \frac{a}{\Sigma a} a s \theta[a-\alpha]$	305[61%]	26[5%]	0.424
16	$a' == \left(\frac{a}{\Sigma a} s - \frac{a}{\Sigma a} a b\right) g \quad b' == s \theta[a-\alpha]$	219[44%]	36[7%]	0.42
17	$a' == \left(s a - \frac{a}{\Sigma a} a b\right) g \quad b' == \frac{a}{\Sigma a} s \theta[a-\alpha]$	398[80%]	261[52%]	0.419
18	$a' == \left(\frac{a}{\Sigma a} s - \frac{a}{\Sigma a} b\right) g \quad b' == s \theta[a-\alpha]$	300[60%]	34[7%]	0.414
19	$a' == \left(s - \frac{a}{\Sigma a} a b\right) g \quad b' == s \theta[a-\alpha]$	243[49%]	33[7%]	0.413
20	$a' == \left(s - \frac{a}{\Sigma a} b\right) g \quad b' == a s \theta[a-\alpha]$	136[27%]	23[5%]	0.412
21	$a' == \left(\frac{a}{\Sigma a} s - \frac{a}{\Sigma a} a b\right) g \quad b' == \frac{a}{\Sigma a} s \theta[a-\alpha]$	227[45%]	37[7%]	0.411
22	$a' == \left(\frac{a}{\Sigma a} s - \frac{a}{\Sigma a} b\right) g \quad b' == \frac{a}{\Sigma a} s \theta[a-\alpha]$	279[56%]	30[6%]	0.41
23	$a' == \left(\frac{a}{\Sigma a} s - a b\right) g \quad b' == \frac{a}{\Sigma a} s \theta[a-\alpha]$	251[50%]	37[7%]	0.409
24	$a' == \left(s - a b\right) g \quad b' == \frac{a}{\Sigma a} a s \theta[a-\alpha]$	218[44%]	26[5%]	0.39
25	$a' == \left(s - a b\right) g \quad b' == s \theta[a-\alpha]$	251[50%]	21[4%]	0.367
26	$a' == \left(s a - \frac{a}{\Sigma a} a b\right) g \quad b' == s \theta[a-\alpha]$	404[81%]	199[40%]	0.366
27	$a' == \left(s - b\right) g \quad b' == \frac{a}{\Sigma a} s \theta[a-\alpha]$	159[32%]	24[5%]	0.358
28	$a' == \left(s - \frac{a}{\Sigma a} b\right) g \quad b' == \frac{a}{\Sigma a} a s \theta[a-\alpha]$	110[22%]	26[5%]	0.333
29	$a' == \left(s - b\right) g \quad b' == a s \theta[a-\alpha]$	186[37%]	15[3%]	0.318
30	$a' == \left(s - b\right) g \quad b' == \frac{a}{\Sigma a} a s \theta[a-\alpha]$	197[39%]	16[3%]	0.312
31	$a' == \left(s - \frac{a}{\Sigma a} a b\right) g \quad b' == \frac{a}{\Sigma a} s \theta[a-\alpha]$	214[43%]	16[3%]	0.294
32	$a' == \left(s - \frac{a}{\Sigma a} b\right) g \quad b' == s \theta[a-\alpha]$	57[11%]	23[5%]	0.28
33	$a' == \left(s - a b\right) g \quad b' == a s \theta[a-\alpha]$	232[48%]	21[4%]	0.278
34	$a' == \left(s a - \frac{a}{\Sigma a} a b\right) g \quad b' == a s \theta[a-\alpha]$	411[82%]	66[13%]	0.162
35	$a' == \left(s a - \frac{a}{\Sigma a} a b\right) g \quad b' == \frac{a}{\Sigma a} a s \theta[a-\alpha]$	402[80%]	70[14%]	0.14
36	$a' == \left(s a - \frac{a}{\Sigma a} b\right) g \quad b' == \frac{a}{\Sigma a} a s \theta[a-\alpha]$	298[60%]	25[5%]	0.127
37	$a' == \left(s a - a b\right) g \quad b' == s \theta[a-\alpha]$	378[76%]	60[12%]	0.115
38	$a' == \left(s a - a b\right) g \quad b' == \frac{a}{\Sigma a} s \theta[a-\alpha]$	364[73%]	71[14%]	0.094
39	$a' == \left(s a - \frac{a}{\Sigma a} b\right) g \quad b' == a s \theta[a-\alpha]$	309[62%]	14[3%]	0.069
40	$a' == \left(s a - a b\right) g \quad b' == \frac{a}{\Sigma a} a s \theta[a-\alpha]$	377[75%]	36[7%]	0.054
41	$a' == \left(s a - a b\right) g \quad b' == a s \theta[a-\alpha]$	392[78%]	40[8%]	0.053
42	$a' == \left(s - \frac{a}{\Sigma a} b\right) g \quad b' == \frac{a}{\Sigma a} s \theta[a-\alpha]$	45[9%]	5[1%]	0.045
43	$a' == \left(\frac{a}{\Sigma a} s a - \frac{a}{\Sigma a} a b\right) g \quad b' == s \theta[a-\alpha]$	335[67%]	12[2%]	0.004
44	$a' == \left(\frac{a}{\Sigma a} s a - \frac{a}{\Sigma a} a b\right) g \quad b' == \frac{a}{\Sigma a} s \theta[a-\alpha]$	331[66%]	13[3%]	0.002

Supplementary Table 3: List of Pareto optimal circuits – model equations and matching scores to behavioral data

	Model	% successful Runs	% runs in HB Convex Hull	Vol(HB⋂M)
1	$\{a' == (\frac{a}{\Sigma a}\frac{s}{b} - a)\,k,\ b' == a\,s\,\theta[a-\alpha]\}$	0.69	0.11	0.527
2	$\{a' == (-1 + \frac{a}{\Sigma a}\frac{s}{b})\,k,\ b' == a\,s\,\theta[a-\alpha]\}$	0.92	0.2	0.524
3	$\{a' == (\frac{a}{\Sigma a}s - b)\,k,\ b' == s\,\theta[a-\alpha]\}$	0.72	0.21	0.502
4	$\{a' == (-a + \frac{s}{b}a)\,k,\ b' == a\,s\,\theta[a-\alpha]\}$	0.94	0.15	0.463
5	$\{a' == (s\,a - \frac{a}{\Sigma a}a\,b)\,k,\ b' == \frac{a}{\Sigma a}s\,\theta[a-\alpha]\}$	0.8	0.52	0.419
6	$\{a' == (-1 + \frac{a}{\Sigma a}\frac{s}{b})\,k,\ b' == s\,\theta[a-\alpha]\}$	0.93	0.24	0.4
7	$\{a' == (-a + \frac{s}{b}a)\,k,\ b' == \frac{a}{\Sigma a}s\,\theta[a-\alpha]\}$	0.96	0.19	0.37
8	$\{a' == (-1 + \frac{a}{\Sigma a}\frac{s}{b})\,k,\ b' == \frac{a}{\Sigma a}s\,\theta[a-\alpha]\}$	0.93	0.3	0.367
9	$\{a' == (s\,a - \frac{a}{\Sigma a}a\,b)\,k,\ b' == s\,\theta[a-\alpha]\}$	0.81	0.4	0.366

Supplementary Table 4: List of top FCD and non-FCD circuits model selection results

model	type	⌗ parameters	AIC	BIC	model disribution
$a' == \left(\frac{a}{\Sigma a}\,\frac{s}{b} - a\right) k$ $b' == a\, s\, \theta[a - \alpha]$	FCD	6	-2.86978	12.7612	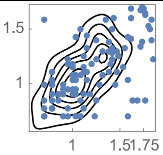
$a' == \left(\frac{a}{\Sigma a}\,\frac{s}{b} - a\right) k$ $b' == \frac{a}{\Sigma a}\, s\, \theta[a - \alpha]$	FCD	6	10.7288	26.3599	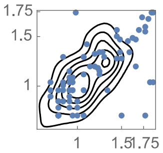
$a' == \left(\frac{a}{\Sigma a}\,\frac{s}{b} - a\right) k$ $b' == s\, \theta[a - \alpha]$	FCD	5	21.0237	34.0496	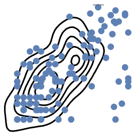
$\alpha]$	FCD	4	80.7422	91.1629	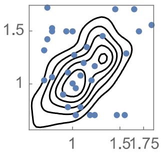

Supplementary References

1. Hart, Y. *et al.* Creative foraging: An experimental paradigm for studying exploration and discovery. *PLOS ONE* **12,** e0182133 (2017).
2. Goldberg, H., Hart, Y., Mayo, A., Alon, U. & Malach, R. Neuronal components of evaluating the human origin of abstract shapes. *bioRxiv* 085902 (2017). doi:10.1101/085902
