# Supplementary material for: Creative exploration as a scale-invariant search on a meaning landscape
Source: Nat Commun. 2018 Dec 21;9:5411. doi: 10.1038/s41467-018-07715-8 (PMC6303308; doi:10.1038/s41467-018-07715-8)
Supplement: Supplementary file 2 — Reporting Summary [file 41467_2018_7715_MOESM2_ESM.pdf]

nature research

Corresponding author(s): Uri Alon

Reporting Summary

Nature Research wishes to improve the reproducibility of the work that we publish. This form provides structure for consistency and transparency in reporting. For further information on Nature Research policies, see Authors & Referees and the Editorial Policy Checklist.

Statistical parameters

When statistical analyses are reported, confirm that the following items are present in the relevant location (e.g. figure legend, table legend, main text, or Methods section).

n/a	Confirmed	
☐	☒	The exact sample size (*n*) for each experimental group/condition, given as a discrete number and unit of measurement
☐	☒	An indication of whether measurements were taken from distinct samples or whether the same sample was measured repeatedly
☐	☒	The statistical test(s) used AND whether they are one- or two-sided<br>*Only common tests should be described solely by name; describe more complex techniques in the Methods section.*
☐	☒	A description of all covariates tested
☐	☒	A description of any assumptions or corrections, such as tests of normality and adjustment for multiple comparisons
☐	☒	A full description of the statistics including central tendency (e.g. means) or other basic estimates (e.g. regression coefficient) AND variation (e.g. standard deviation) or associated estimates of uncertainty (e.g. confidence intervals)
☐	☒	For null hypothesis testing, the test statistic (e.g. *F*, *t*, *r*) with confidence intervals, effect sizes, degrees of freedom and *P* value noted<br>*Give P values as exact values whenever suitable.*
☒	☐	For Bayesian analysis, information on the choice of priors and Markov chain Monte Carlo settings
☒	☐	For hierarchical and complex designs, identification of the appropriate level for tests and full reporting of outcomes
☐	☒	Estimates of effect sizes (e.g. Cohen's *d*, Pearson's *r*), indicating how they were calculated
☐	☒	Clearly defined error bars<br>*State explicitly what error bars represent (e.g. SD, SE, CI)*

Our web collection on statistics for biologists may be useful.

Software and code

Policy information about availability of computer code

Data collection	Behavioral data was collected using an online game developed in processing.js. MRI data was acquired on a 3 Tesla Trio Magnetom Siemens scanner at the Weizmann Institute of Science.
Data analysis	We used Mathematica 11.3 for analysis of behavioral data and circuit dyanmics analysis, and Brain Voyager QX 2.6 software package for brain imaging data analysis.

For manuscripts utilizing custom algorithms or software that are central to the research but not yet described in published literature, software must be made available to editors/reviewers upon request. We strongly encourage code deposition in a community repository (e.g. GitHub). See the Nature Research guidelines for submitting code & software for further information.

Data

Policy information about availability of data

All manuscripts must include a data availability statement. This statement should provide the following information, where applicable:

- Accession codes, unique identifiers, or web links for publicly available datasets
- A list of figures that have associated raw data
- A description of any restrictions on data availability

Provide your data availability statement here.

April 2018

Field-specific reporting

Please select the best fit for your research. If you are not sure, read the appropriate sections before making your selection.

☐ Life sciences ☒ Behavioural & social sciences ☐ Ecological, evolutionary & environmental sciences

For a reference copy of the document with all sections, see nature.com/authors/policies/ReportingSummary-flat.pdf

Behavioural & social sciences study design

All studies must disclose on these points even when the disclosure is negative.

Study description	The study combines behavioral measures of people in an online game, and brain activity data from a separate group of participants. Both data sets are quantitative.
Research sample	Behavioral data was gathered from the Hebrew University study pool (undergraduates) of the psychology department. Brain activity data was recruited from the Weizmann Institute study pool of the neuroscience department.
Sampling strategy	Sampling was random. Sample sizes were not determined on the basis of a pilot experiment but were predetermined before the experiments begun. Both behavioral and brain data analysis show narrow CI ranges, indicating a sufficient number of participants was gathered for the statistical tests performed in this study.
Data collection	Behavioral data was collected using a computer on which subjects played the online game. Brain data was collected through scanning subjects in a 3 Tesla Trio Magnetom Siemens scanner at the Weizmann Institute of Science.
Timing	Behavioral data was collected on 2012. Brain activity data was collected on 2014.
Data exclusions	In the brain activity data, 2 subjects did not participate in the full setup of the experiment and were removed from analysis.
Non-participation	No participants dropped/declined to participate in the study.
Randomization	Allocation of participants was random.

Reporting for specific materials, systems and methods

Materials & experimental systems

n/a	Involved in the study
☒	☐ Unique biological materials
☒	☐ Antibodies
☒	☐ Eukaryotic cell lines
☒	☐ Palaeontology
☒	☐ Animals and other organisms
☐	☒ Human research participants

Methods

n/a	Involved in the study
☒	☐ ChIP-seq
☒	☐ Flow cytometry
☐	☒ MRI-based neuroimaging

Human research participants

Policy information about studies involving human research participants

Population characteristics	For the behavioral data: Undergraduate students at the Hebrew university (54 females, 46 males, age 20–49, mean(±std) = 25(±4)), took part in the experiment.<br>For Brain data: Seventeen healthy right handed subjects (ages 28 ± 3.8, 10 females) participated in the fMRI experiments.
Recruitment	For the behavioral data, participants were recruited from the Hebrew University study pool. For the brain data, participants were recruited from the Weizmann Institute neuroscience department study pool.

Magnetic resonance imaging

Experimental design

Design type	We used task related, block design.
Design specifications	29 blocks, 9 sec each block and 9 sec intervals of rest in between.
Behavioral performance measures	Participants performed a color classification task during the scan but no behavioral measures were gathered for this study

Acquisition

Imaging type(s)	Functional
Field strength	3T
Sequence & imaging parameters	Functional images of blood oxygenation level dependent (BOLD) contrast comprising of 46 axial slices were obtained with a T2*-weighted gradient echo planar imaging (EPI) sequence (3 × 3 × 3 mm voxel , TR = 3000 ms, TE = 30, flip angle = 75°, FOV 240 mm) covering the whole brain.
Area of acquisition	Whole brain scan

Diffusion MRI ☐ Used ☒ Not used

Preprocessing

Preprocessing software	Brain Voyager QX 2.6 software package for brain imaging data analysis.
Normalization	Linear transformation into AC-PC, Talairach normalized brain space.
Normalization template	Anatomical images of each subject were acquired in order to incorporate the functional data into the 3D Talairach space (Talairach and Tournoux, 1988) using 3D T1- weighted images with high resolution.
Noise and artifact removal	The first 7 images of each functional scan (including the extra initial block and rest) were discarded. Functional scan preprocessing included 3D motion correction and filtering out of low frequency noise (slow drift), and spatial smoothing using an isotropic Gaussian kernel of 6 mm full-width-half-maximum (FWHM).
Volume censoring	TRs containing motions larger than 1 mm were discarded from analysis. This occurred in one scan of one subject.

Statistical modeling & inference

Model type and settings	Parametric GLM analyses was conducted based on shapes meaning, in a multi-subject, random effect analyses. Each block of shapes received a weight according to its meaning score which was represented in the model as differential amplitude of the BOLD signal.
Effect(s) tested	The lateral occipital complex (LOC) was the main brain region showing spontaneous selectivity to shapes' meaning, even during a low level task (color classification). LOC activity was parametrically correlated to the shape's meaning score. For the fitting of different decay functions for the meaning LOC was defined independently using an external localizing task.

Specify type of analysis: ☐ Whole brain ☐ ROI-based ☒ Both

Anatomical location(s)	LOC- relevant voxels were defined functionally and bilaterally from a second fMRI scan of the participants. To define the LOC, a contrast between two types of shape groups was done; abstract shapes created by human players ('human') vs. abstract shapes created by a random walk algorithm ('random').
Statistic type for inference (See Eklund et al. 2016)	Voxel-wise
Correction	We performed both Monte Carlo and T-max correction analyses.

Models & analysis

n/a	Involved in the study
☒	☐ Functional and/or effective connectivity
☒	☐ Graph analysis
☒	☐ Multivariate modeling or predictive analysis
